# Facial micro-movements as a proxy of increasingly erratic heart rate variability while experiencing pressure pain

**DOI:** 10.3389/fnins.2026.1702124

**Published:** 2026-04-01

**Authors:** Elizabeth B. Torres, Mona Elsayed

**Affiliations:** 1 Sensory Motor Integration Laboratory, Psychology Department, Rutgers the State University of New Jersey, New Brunswick, NJ, United States; 2 Rutgers University Center for Cognitive Science, Rutgers the State University of New Jersey, New Brunswick, NJ, United States; 3 Center for Biomedicine Imaging and Modelling, Computer Science Department, Rutgers the State University of New Jersey, New Brunswick, NJ, United States

**Keywords:** facial micro-movements, HRV, pain, proxy parameter, stochastic process

## Abstract

**Introduction:**

The sensation of pain varies from person to person. These patterns of individual variation are difficult to capture using coarse subjective self-reports. However, they are important when prescribing therapies and tailoring them to each person’s own sensations. Pain can be experienced differently by the same person and can fluctuate based on context; yet, most analyses treat the problem with a one-size-fits-all model.

**Methods:**

In this work, we introduce a series of assays to assess pressure pain across tasks with different motoric and cognitive demands, in relation to a resting state. In a cohort of healthy individuals, we examine pain-free *vs*. pain states at rest, during drawing with heavy cognitive demands, during pointing to a visual target, and during a grooved peg task, such as inserting a grooved key into a matching keyhole. We adopt a standardized data type called micro-movement spikes (MMS) to characterize the biorhythmic activities of facial micro-expressions and the micro-fluctuations in the heart’s inter-beat interval timings.

**Results:**

Using the MMS peaks, we find that the continuous Gamma family of probability distribution functions best fits the frequency histograms of both the facial and heart data. Furthermore, we find that the Gamma shape and scale parameters in both signals span a scaling power law whereby, as the noise-to-signal ratio (Gamma scale parameter) increases, so does the randomness of the stochastic process. We find that as the heart IBI becomes more erratic (noisier and more random), the facial ophthalmic region also increases in noise and randomness, with higher linear correlation for tasks requiring haptic feedback (*R*^2^ 0.84) and lower correlation for tasks requiring greater cognitive and memory loads (*R*^2^ 0.77).

**Conclusion:**

Increases in transfer entropy show that recent past activity (~167 ms back) of the heart IBI and facial data combined lower the uncertainty in predicting the present ophthalmic facial activity, suggesting that this facial region may serve as a proxy for the increasingly dysregulated heart. These results have implications for the detection and monitoring of pressure pain.

## 1 Introduction

The sensation of physical pain can be experienced through different afferent channels and reach a conscious level, as one attempts to describe it amidst the constant flow of kinesthetic reafference generated by movements and biorhythms throughout the body. How can the brain’s self-generated biorhythms differentiate pressure pain in relation to the body’s resting state and produce a conscious recollection of the experience? We know from the literature that reports of pressure pain are often associated with individuals who experience chronic pain (Amiri et al., 2021), and these individuals tend to have a lower pressure pain threshold than healthy controls (Kim et al., 2019). Often, these reports are provided by the person while at rest. However, in the context of activities of daily living and natural situations where pain is present or has been experienced, it may be pertinent to ask whether biorhythms from self-generated movements across various tasks could differentiate between the tasks before and during the experience of pain at a level high enough to reach conscious recollection.

One channel that may be amenable to discern intensity in physical body experiences and differentiating pain sensations amidst natural motions embedded in naturalistic tasks could be the face. Facial micro-expressions can transpire largely beneath awareness and yet help us automatically differentiate transitions across behavioral states, which can now be captured with simple means, such as a webcam or a cell phone app using the phone’s camera (Torres et al., 2025). With the advent of new models from computer vision, it is possible to convert video of the face into a face grid and register minute fluctuations in facial micro-motions. Using new algorithms from our lab (Torres, 2017a,b, 2019), one can extract movement-relevant information from very brief (5 s long) videos. Such methods offer the advantage of easy and frequent sampling across a variety of contexts, including the comfort of one’s home (Elsayed et al., 2025). If we measure other biorhythms in tandem with those from the face, we can also characterize internal physiological activities that indicate possible states of distress and anxiety, such as those present during the fight-or-flight response related to the heart’s biorhythms (Elsayed and Torres, 2023; Elsayed, 2024). Measuring heart rate variability (HRV) across different states of pain, relative to rest, could then provide additional information to complement facial data and approach pain differentiation through multiple reafferent channels. The autonomic channels are particularly suitable for this, as the field of cardio-physiology has made significant strides in analyzing HRV data (e.g., (Lobdell et al., 2023; Palermo et al., 2023; Weng et al., 2024)). Their results can serve as ground truth to derive, using facial video data, highly scalable, unobtrusive means that offer proxy signals of pain sensation as a metric of autonomic dysregulation. Recent work involving advanced machine learning algorithms and multiple open databases offers new insights into the automated classification of pain states across facial video data integrated with other biorhythmic activities (Gkikas et al., 2025).

In this work, we examine HRV and facial micro-expressions before and during the lingering sensation of pain experienced while performing different tasks with different levels of kinesthetic reafference and cognitive/memory demands. From purely continuous movement speed feedback to movement speed feedback combined with haptic- and pressure-based feedback, we characterize levels of noise in both HRV and facial micro-movements and quantify the degree to which some tasks better differentiate the experienced pain sensation than others. We find that shifts in the noise patterns of the heart and face are linearly correlated in tasks with haptic–pressure feedback demands. These tasks also result in higher pain differentiation than tasks with additional cognitive/memory demands, suggesting that cognitive/memory demands could be used as pain distractors. Furthermore, we found evidence that recent past signals of heart inter-beat interval timings—during more erratic pain activity—tend to increase the certainty of predicting present facial micro-motions. We propose that moment-to-moment micro-fluctuations in facial micro-movements during heart dysregulation may serve as a proxy for such erratic autonomic patterns, helping us differentiate individualized levels of pain tolerance across the population. The implications of this research for the scalability and design of pressure-pain treatments are discussed.

## 2 Materials and methods

### 2.1 Participants

**Figure 1:**
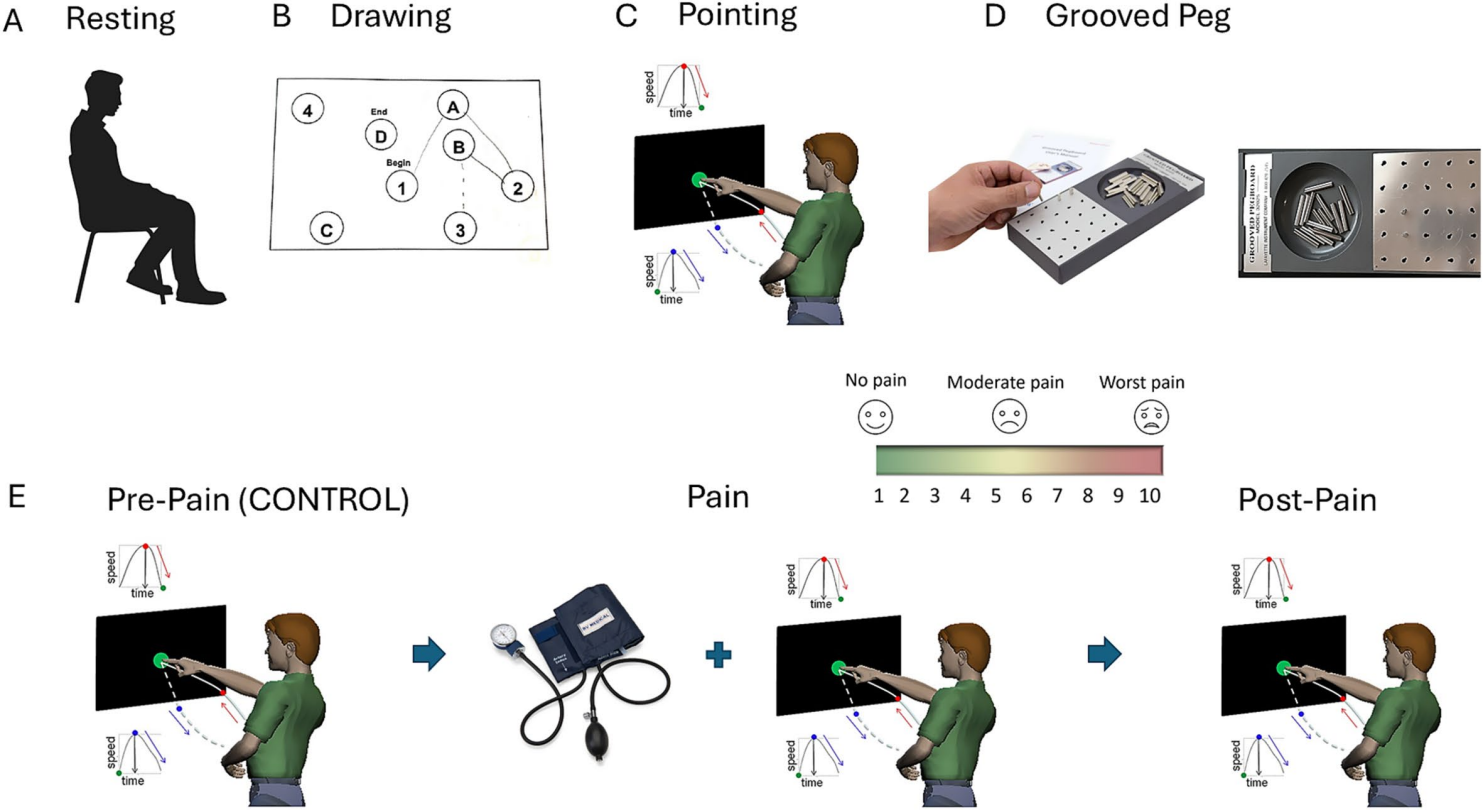
Different tasks in an assay to measure the effects of pressure pain. **(A)** Resting task where the person sits and is instructed to remain as still as possible. **(B)** Drawing task where the person draws a line connecting digits and letters in a specified order (1 A, 2 B, etc.) from beginning to end. **(C)** Pointing task, where the person is asked to point to a target (the full loop of forward motion to the target and backward motion toward rest). The second backward segment occurs spontaneously, without instruction, while the first motion toward the target is instructed. **(D)** The grooved peg task where the person picks up a grooved peg and aims it at the grooved hole on the board to insert it in the appropriate orientation. This task requires orienting the grooved peg to match the grooved hole, similar to opening a door by inserting a key into the lock. **(E)** Experimental assay to probe the control condition *vs*. the pain condition. For each task (using the pointing task as an example), the person performs the task normally, without any pain (control case). A blood pressure cuff is then placed on the non-dominant arm (the arm that is not performing the task) and inflated to 200 mmHg for the entirety of the task to ensure lingering discomfort that produces the sensation of physical pressure pain. The person performs the task under pain (pain condition), and lastly, the person performs the task after the pain disappears (post-pain condition).

In this study, 45 healthy, neurotypical adults (27 female and 18 male) of college age were recruited to participate via flyers, advertisements, or through the Rutgers Human Research Pool system. All participants provided informed consent, which was approved by the Rutgers University Institutional Review Board (Study ID #Pro2019000615). Wearable sensors and webcams were used to record biorhythmic activity across the face, heart, and body while individuals performed each task separately. In a subset of 21 individuals, we recorded concurrent heart and facial activities and analyzed their performance. Figures 1A–D depicts the tasks in the order performed, while Figure 1E provides the assay to elicit responses across conditions. All tasks were subject to this assay, comprising an adaptation phase (denoted pre-pain or control interchangeably throughout the paper) and a pain phase in which the person experienced physical pressure pain. This work is part of a PhD thesis published in 2024 (Elsayed, 2024).

### 2.2 Tasks and assays

Four tasks were utilized in the study to assess the effects of pain on physiological and motor–cognitive functioning: Resting, Drawing, Pointing, and Peg. The tasks were chosen to evoke movements in different contexts, according to different levels of feedback and cognitive/memory demands acting as possible distractors from the pain. Both the Drawing and Grooved Peg Tasks (referred to as the Peg Task from here onward) had a haptic component mediated by pressure. However, the Drawing Task had additional memory and cognitive demands that we will discuss below. The Resting, Drawing, Pointing, and Peg tasks were each performed in consecutive order during three different conditions. The first condition (Pre-Pain) served as the control, where the participant performed each task without any pain induction. The Pain condition served as the experimental condition, where pressure pain was induced via a blood pressure cuff on the non-dominant arm that was not used to perform the tasks. The last condition (Post-Pain) served to assess any aftereffects of pain induction or lingering sensations of pain as it dissipated. Each task was performed consecutively (Drawing, Pointing, Peg) under each of the three conditions, except for the Resting Task, which was performed only during the Pre-Pain (control) and Pain conditions. In this way, the adaptive nature of the movement and the pain experience could be assessed through continuous adaptive processes.

*Task 1* is the Resting Task. Participants were instructed to sit still and comfortably at rest for 3 min and to avoid excessive movement (Figure 1A). This task of sitting still is quite difficult, as the human body, even when seemingly at rest, is in constant motion. Breathing, fidgeting, and, in some cases, involuntary tics are common across the population and often exacerbated by psychotropic medications (Torres and Denisova, 2016); yet these motions transpire largely beneath awareness. By sampling the bodily micro-movements at rest while instructing the person to remain still, we are, in fact, assessing the level of volitional control that the person’s system has over involuntary motions.

*Task 2* is the Drawing Task (Figure 1B). This task is a component of the Montreal Cognitive Assessment (MoCA) (Nasreddine et al., 2005) used to evaluate the person’s cognitive and memory abilities as the task goals increase in complexity. With their dominant hand, the participant uses pen and paper to create a series of trails connecting letters and/or numbers. The numeric portion involves drawing a connected path across digits in a random order. For example, from 1 to 10, the sequence could be 2, 5, 3, 7, 4, 1, 9, 10, 6, 8, spread out across the board, and the person would have to join the numbers in order—1 followed by 2, followed by 3, *etc.*, all the way to 10—by continuously tracing the path of the pen on the piece of paper. This action provides continuous feedback from the surface of the table based on the pressure that the hand exerts on the pen and the pen on the table’s surface. Such continuous feedback offers a type of haptic information that the brain needs to monitor and modulate the applied pressure (needed to keep the pen moving against the surface). This occurs simultaneously with the cognitive load required for ordering the digits on the paper. The task also engages spatial memory capacity to retain the correct order already visited and the upcoming digit at a different location on the paper. An additional layer of cognitive load arises when the person must sort an alphanumeric string as the letters A, B, C, D, …, etc., are added to the numbers. In the alphanumeric portion, 1A, 2B, 3C, …, etc., must be considered in the proper order to trace the paths connecting the digits and letters randomly located across the paper. The subject’s performance on this task is judged in terms of the time, in seconds, required to complete all trails. Often, the timing of these traces increases when the alphanumeric case is included in the assay (Ryu et al., 2019; Vero, 2025), evidencing (among other kinematic parameters) that the cognitive and memory loads are significantly higher in alphanumeric mode than in the simpler digit mode. This Drawing Task is an example of the intersection between cognition and motor control (Vero, 2025).

*Task 3* is the Pointing Task (Figure 1C). In this task, participants extended their dominant arm to point to a visual target and then brought their arm back to rest, repeatedly, 50 times. This Pointing Task involves a forward, goal-directed phase where the hand is deliberately aimed at the visual target at one’s own pace (comfortable speed). This motion is followed by an uninstructed, spontaneous backward retraction of the hand, bringing it back to rest without the participant even realizing that the motion is taking place. The backward motion is quite automatic. Two fundamental types of movements are coordinated and sequenced throughout the task (Torres, 2011). Previous work with the deafferented case study Ian Waterman (Torres et al., 2014) and autistic participants (Torres et al., 2013) supports the choice of this task to probe the sense of kinesthetic reafference and the role of noise signatures in motor control and coordination. Here, we were interested in the differentiation of this task in Pain-free *vs*. Pain conditions.

*Task 4* is the Peg task (Figure 1D). During the Peg task, the participant uses their dominant hand to insert 25 keyed pegs into the randomly positioned slots of the Purdue Grooved Pegboard at their own pace (Lafayette, IN). This Peg Task has a transport phase from the hand resting position to the grooved peg reservoir, followed by a phase of picking up the grooved peg (sensing through haptic feedback the groove of the peg and applying adequate pressure to hold it against gravity), then lifting it to transport it to one of the grooved holes on the pegboard and successfully inserting it into the groove-matching hole. This task has a strong biomechanical compliance component as well as a strong cognitive component. The biomechanical component requires aligning the arm and hand according to physical space constraints to facilitate the execution of the task. The cognitive component involves performing multiple coordinate transformations from visual to proprioceptive spaces before the motion initiates and continuously adjusting them during the execution of the action. A solution to Bernstein’s degrees-of-freedom problem is needed here (Bernstein, 1967) to translate the groove orientation correctly into the proper configuration of the arm’s rotational joints, resulting in optimal hand orientation (Torres and Zipser, 2002). Facilitating the alignment of the grooved peg that the hand holds with the grooved hole on the pegboard is like successfully inserting a key in a lock, e.g., to open a door. Continuous haptic feedback can then help the brain determine the proper coordinate transformations necessary to transport and accurately fit the peg in the hole according to the groove. Modulating the linear speed during the translation of the arm’s segments, including the hand, and monitoring the angular speed during the rotations of the arm’s joints occur simultaneously in such a goal-directed task during its orientation-matching phase (Torres and Zipser, 2004). Part of our hypothesis is that because of their complexity, these mental operations may distract the mind from the underlying pain sensation as the person attempts to solve the task correctly. Multiple attempts/errors are allowed since the goal of our study was to evaluate the nervous system pre- and during-pain, rather than to evaluate the accuracy of the performance. Together, the Pointing, Grooved Peg, and Drawing tasks constitute our assay to digitally measure pre- and during-pain sensations relative to the baseline resting state.

### 2.3 Sensors and data extraction

We used video cameras and wearable sensors to record the facial and bodily biorhythmic data, respectively. Below we describe the data acquisition process to motivate the data analysis.

#### 2.3.1 Facial data acquisition

**Figure 2:**
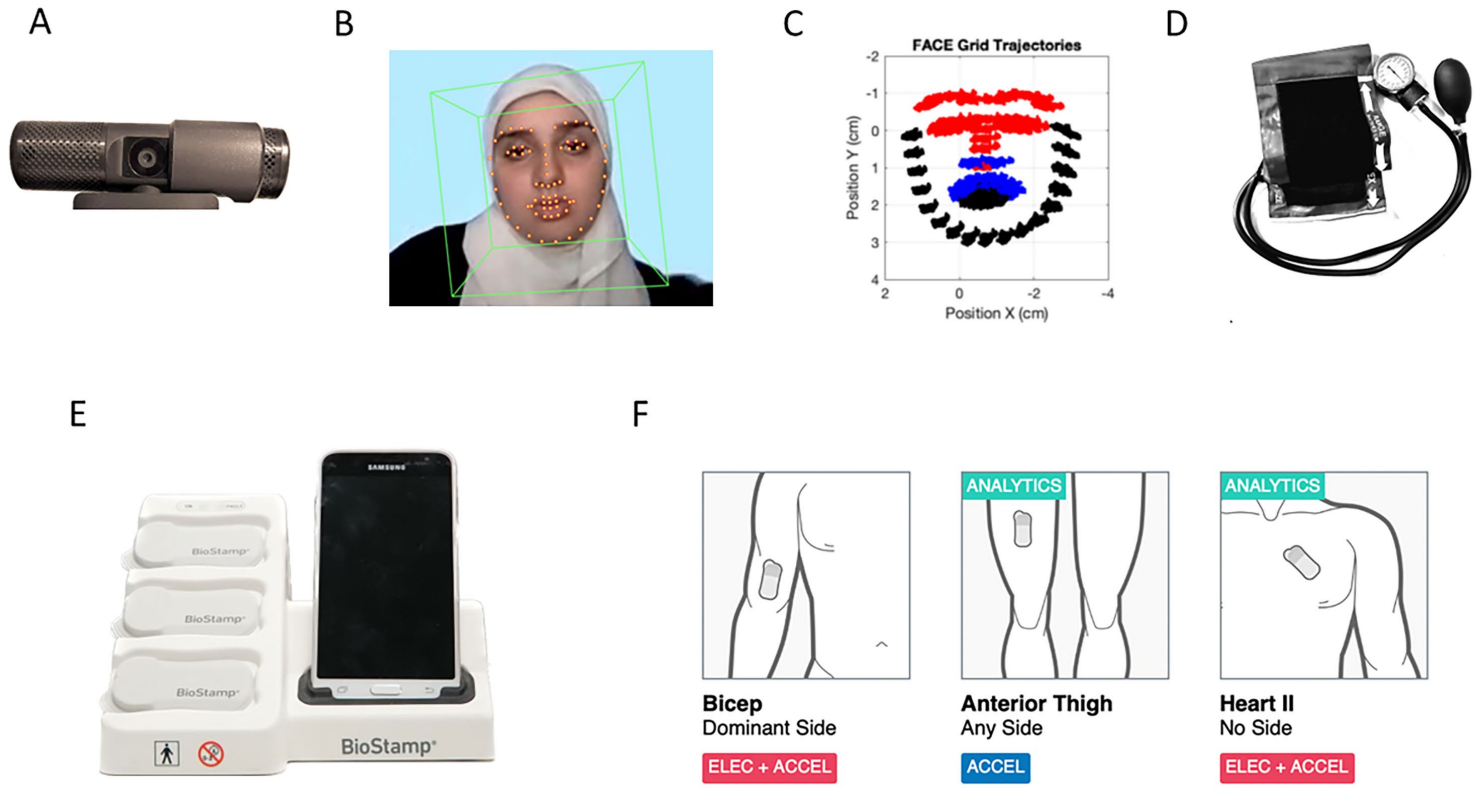
Data acquisition tools. **(A)** A webcam collects video of the participant for the full duration of the task. **(B)** OpenFace software is used to estimate the facial grid from the video of one of the authors (Dr. Elsayed), along with the 3D gaze and head orientation, saving action units responsible for the micro-motions of the facial muscles. **(C)** Trigeminal subdivision based on the facial nerves innervating three main regions: V1 (ophthalmic), V2 (maxillary), and V3 (mandibular). The facial grid is subdivided into V1 (red), V2 (blue), and V3 (black) subregions to study the stochasticity of the micro-motions. **(D)** Blood pressure cuff used to evoke the sensation of pressure pain in the non-performing arm. **(E)** The MC10 Biostamp sensors and phone used to collect time series data by registering different biorhythms. **(F)** Locations on the body where the Biostamp sensors were placed to co-register data from the biceps, anterior thigh, and heart (image acquired via registered study on the MC100 Biostamp-n-Point Cloud system, now acquired by Medidata).

A webcam (as depicted in Figure 2A, Logitech, 30 Hz) was used to acquire brief videos across the four tasks described above and for the pre- and during-pain conditions. The camera was set up about one foot away from the participant and centered at their eye level so that their face was visible throughout the experiment. The regions of interest have been defined in a different publication (Torres et al., 2025), spanning 26 points of the grid V1 (17:30 33 36–47); 17 points of the grid, V2 (31 32 34 35 48 49:53 54 68:64); and 25 points of the grid, V3 (0:16 55:59 65:67).

The participant’s face projected its natural expressions while engaging in the tasks. We did not ask the participant to make any specific facial expressions; rather, we recorded their face as the tasks and assays unfolded. After collecting the video, we ran OpenFace (see Notes 1) and retained the facial grid, 3D gazes, and action units, as shown in Figure 2B. We then used the trigeminal nerve (cranial nerve V) regions to identify three subregions of the face (ophthalmic, maxillary, and mandibular), which we designate as V1 in red, V2 in blue, and V3 in black, respectively. These are illustrated in Figure 2C. The subregions are important for sensing movement reafference throughout key facial points involved in social and emotional communication (e.g., the eyes, ears, mouth, lips, tongue, and mandibular regions) and offer the potential to differentiate activities according to levels of pain and task complexity.

#### 2.3.2 Heart signal acquisition and pain induction procedure

We measured the activity of the heart using the MC10 Biostamp-nPoint system (Lexington, MA), placing one sensor with four electrodes in the lead II position on the chest. To induce pressure pain, a blood pressure cuff (a standard tourniquet, as shown in Figure 2D) was wrapped around the non-dominant arm of each participant and inflated to 200 mmHg for the duration of each task (about 1–3 min) to create a lingering sensation of pain. This method serves as a modified version of the submaximal effort tourniquet test (Sternbach et al., 1977), which has been shown to emulate pathological pain (Pertovaara et al., 1984). The Biostamp sensors are depicted in Figure 2E, along with the smartphone that enables time stamping of the signals, labeling, and automatic uploading to the cloud. Figure 2F shows the body locations where the Biostamp sensors were affixed. Adhesive gel tapes were used to help register the electrical activity from the muscles (electromyography, EMG) and the heart (electrocardiography, ECG). In this paper, we focus on the ECG signals and reserve the other data for a different publication.

### 2.4 FACE data analysis

**Figure 3:**
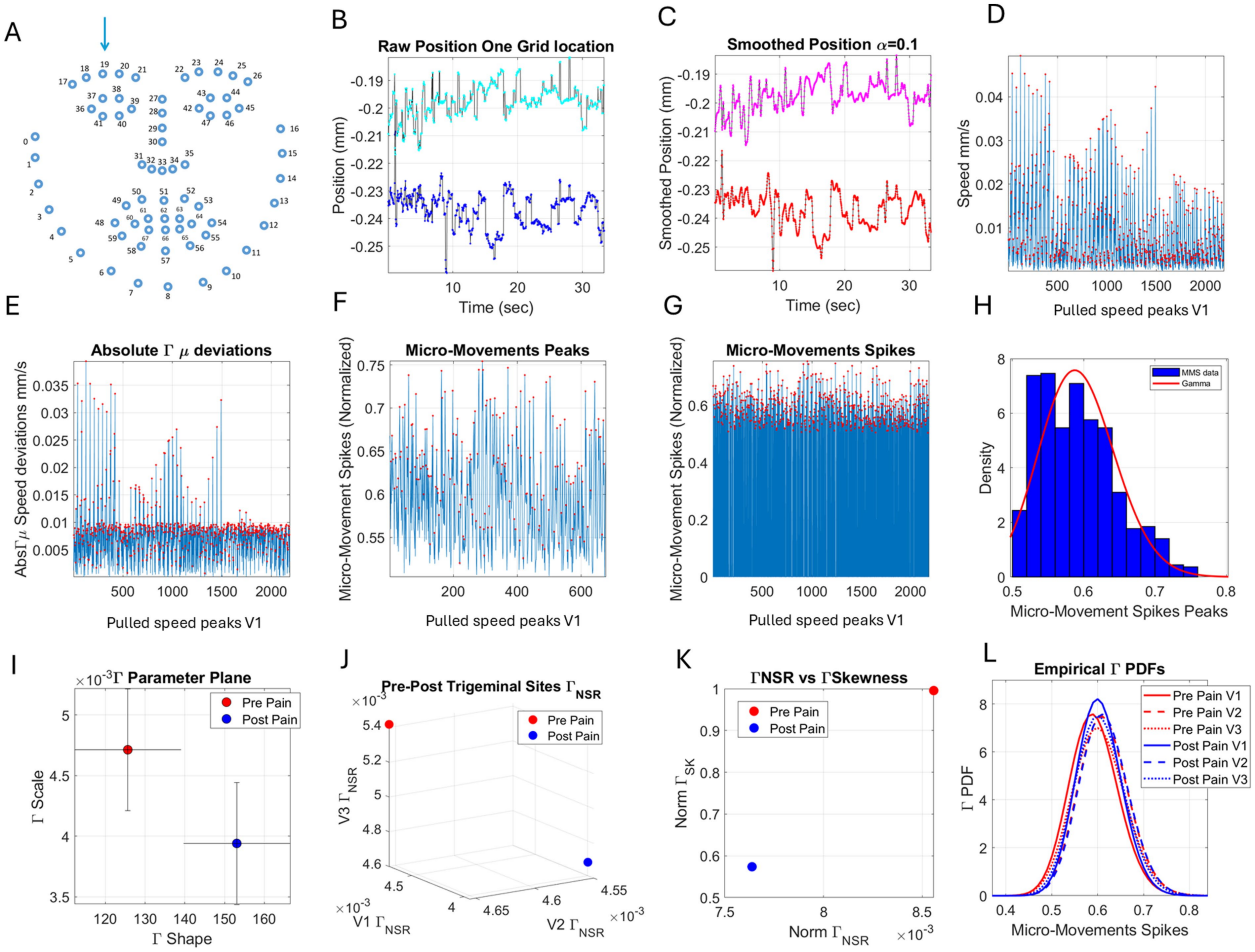
Analytical pipeline of facial biorhythms. **(A)** Face grid mapped (indexed) across regions: ophthalmic V1, maxillary V2, and mandibular V3. **(B)** Sample XY-position traces of one grid point (19 in **(A)**) recorded for 35 s. (C) Smoothed trajectories. (D) Scalar speed profiles from 26 sites in region V1. **(E)** Absolute speed deviations from the empirically estimated mean (from the red peaks). **(F)** Locally scaling allometric effects using Equation 1, due to anatomical disparities in lengths, impacting speed ranges across participants. **(G)** Full micro-movement spikes preserving the frame of each original speed peak. **(H)** Frequency histogram of the MMS peaks. **(I)** Gamma parameter plane used to represent the empirically estimated Gamma PDFs as the shape and scale parameters with 95% confidence intervals for each parameter. **(J)** Parameter space spanned by the Gamma NSR (scale parameter) of each PDF thus estimated for each face region. **(K)** Parameter plane defined by the norms of the Gamma NSR vector spanned by the V1, V2, and V3 NSRs and the corresponding Gamma PDF skewness. **(L)** Empirical Gamma PDFs for each region and condition (control *vs.* pain).

**Figure 4:**
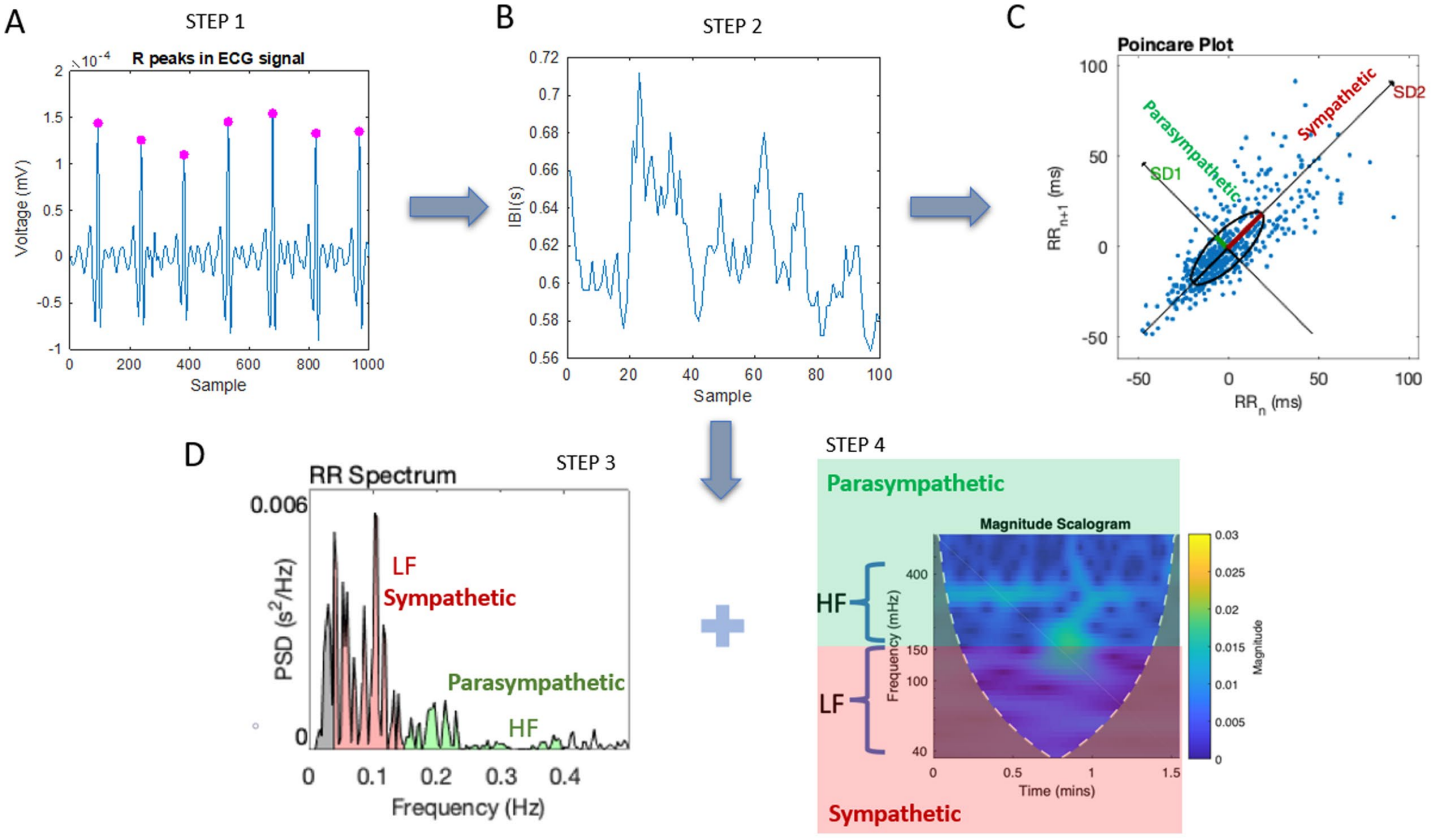
An analytical pipeline for pre-processing the cardiac data. **(A)** Upon filtering noise and detrending the ECG data, the R-peaks are localized, and in **(B)**, the temporal distances between the peaks are obtained. This produces the RR-peaks series data or inter-beat interval times series (IBI). **(C)** The temporal domain is assessed by obtaining the Poincaré plot, a shifted version of the time series one step forward along the *y*-axis and the current time version along the *x*-axis. The scatter is fitted by an ellipse, and the principal axes are examined as SD1 (variability associated with the parasympathetic system) and SD2 (variability associated with the sympathetic system). These parameters are then examined in relation to noise regimes from the empirically estimated micro-movement spikes (micro-peaks) distributions. **(D)** In this step, we perform frequency domain analysis using power spectral decomposition techniques and obtain the low- and high-frequency bands for examination of the sympathetic and parasympathetic regimes associated with lower and higher frequencies, respectively. A magnitude scalogram can then be used to visualize the data as frequency over time with power information (color bar representing the magnitude/power levels).

We focus on video data acquired from the face and the inter-beat intervals obtained from the ECG, providing the R–R peaks. Figure 3 presents the analytical pipeline for the face data, while Figure 4 provides it for the heart data. We reserve the eye gaze and head orientation data from the face videos for a different publication.

The face grid consists of 68 points indexed across the face, as shown in Figure 3A, while considering the trigeminal areas V1, V2, and V3 depicted in Figure 2C. The analytical methods have been previously described (Torres et al., 2025). Here, we briefly summarize them. The positional pixel trajectories of facial movements were obtained using OpenFace estimation (Baltrušaitis et al., 2016) (and see Note 1), from brief videos recorded during the experiment (e.g., 35 s are shown in Figure 3B from the location indexed as #19 in Figure 3A). This positional trajectory along the *x* and *y* coordinates was smoothed in Figure 3C using in-house developed code employing spline interpolation routines from MATLAB (version R2023b). The speed profiles derived from differentiating the smoothed positional trajectories were used to construct the velocity field at each indexed point of each region of the face. The speed magnitude (pixels/s) was then obtained at uniformly spaced time intervals sampled at 30 Hz, using the Euclidean norm. For example, Figure 3D shows the speed profiles of the 26 points comprising the ophthalmic region V1 pooled together in the order (17:30 33 36–47). We note here that we are examining the moment-by-moment fluctuations in amplitude rather than the times at which these fluctuations occurred. For each participant and condition, we use the same landmarks to aggregate the data corresponding to each region (V1, V2, V3).

The peak speed was determined at the points where the slope of the temporal speed profile (the speed curve) changed from positive to negative. Likewise, the valleys surrounding each peak were the points where the slope of the curve changed from negative to positive. We grouped the speed peaks in a frequency histogram and used maximum likelihood estimation to determine which continuous family of probability distribution functions best fit the histogram, with 95% confidence. We tried several distribution families, including the normal, lognormal, Weibull, and Gamma.

Consistent with our previous work (Torres et al., 2013, 2018, 2025), the continuous Gamma family of probability distribution functions was the best fit in an MLE sense. We then obtained the first moment (the mean) and subtracted it from each point in the original time series. This provided the distribution of absolute deviations from the empirical Gamma mean.

The speed profiles pooled across 26 grid points in V1 are shown in Figure 3D. The positive (absolute) speed deviations from the empirically estimated Gamma mean are shown in Figure 3E. These positive deviations from the mean were normalized by Equation 1 to locally scale out allometric effects due to anatomical disparities involving facial motion path length, which impact speed, our parameter of interest. Accordingly, for higher speed values, on average, the moment-to-moment fluctuations in facial speed will be lower than for slower speed values. This time series of normalized fluctuations from the empirical mean is termed the micro-movement spikes (MMS). The data at zero value corresponds to the mean speed level. The spikes span the real number range [0, 1] but can also be treated as a binary time series spike train using the rate parameter, with physiological relevance quantified through information theoretical metrics (Torres, 2017b):


$$\mathrm{MM}S_{\text{FACE SPEED}}=\frac{\mathrm{Pea}k_{\text{FACE SPEED}}}{\mathrm{Pea}k_{\text{FACE SPEED}}+\mathrm{Avr}g_{\min\;\text{to}\;\min}}$$ (1)

As mentioned above, in Equation 1, the local *Peak* term refers to each point in the time series where the slope of the waveform changes from positive to negative. The local neighboring minima (the valleys surrounding the local peak) provide the bounding values to obtain the local averages of speed used in the normalization (*Avrg*). In this sense, small speed averages lead to higher MMS fluctuations away from the empirical mean.

The peaks of the absolute deviations were normalized, as explained above, to eliminate the allometric effects due to disparate anatomical lengths of the faces across participants. These scaled peaks are shown in Figure 3F for peaks deviating from the empirical Gamma mean. Figure 3G shows the micro-movement spikes in their entirety, preserving the frames where they occurred. Note that 0-values represent the mean motion, while non-zero values are the deviations from the mean. Figure 3H provides an example of a histogram from the micro-movement spikes (peaks) and the best fitting probability distribution function (which also resulted in the continuous Gamma family, according to MLE).

A random variable X that is Gamma-distributed with shape *a* and scale *b* is denoted by:


X∼Γ(a,b)=Gamma(a,b) (2)

The probability density function using the shape-scale parameterization is:


$$f(x,a,b)=\frac{x^{a-1}e^{-x/b}}{b^{a}\Gamma (a)}\;\;\text{for}\;\;x>0\;\;\text{and}\;\;a,b>0$$

Here, Γ(a) is the Gamma function evaluated in *a*.

Figure 3I shows the Gamma parameter plane with two distribution samples from the control condition (pre-pain) and the pain condition. These points represent the shape and scale parameters of the Gamma PDF estimated for each case, corresponding to a region of the face. Points are represented by 95% confidence intervals. Figure 3J displays the points for each face region along the V1 (*x*-axis), V2 (*y*-axis), and V3 (*z*-axis), separating the control from pain distribution values of the Gamma PDF’s noise-to-signal ratio (NSR).

The Gamma NSR is the scale parameter denoting the dispersion of the distribution. Given the Gamma shape *a* and the Gamma scale *b*, the NSR or variance-to-mean ratio, which is the Gamma variance $\sigma^{2}=a\cdot b^{2}$ divided by the Gamma mean μ=a⋅b , gives the scale parameter *b* as our predictor of the NSR.

We have found a strong linear correlation between the log–log of these two parameters along the logarithmic 10-based scale across the human lifespan (Torres et al., 2013). As the Gamma NSR (scale parameter) decreases, the Gamma shape increases toward the Gaussian regime, moving away from the memoryless random exponential regime. This occurs as maturation takes place in the sensory–motor systems. However, such motor control milestones fail to appear in autism and revert to high Gamma NSR and random exponential regimes in neurodegenerative (Ryu et al., 2019; Torres et al., 2014) or neuropsychiatric disorders like schizophrenia (Nguyen et al., 2016). This result has extended to multiple biorhythms of the nervous system, enabling us to reduce the two parameters to one of interest; given one parameter, we can infer the other with high certainty. Accordingly, we examine this scaling power law for the face and heart data. Further, we analyze the Gamma NSR for each facial region and plot it as shown in Figure 3J, creating a vector representation where the points are measured from the (0, 0, 0) origin using the Euclidean norm. Based on this representation of the Gamma NSR across the facial regions, we then obtain the scalar value summarizing the facial noise, which also gives insight into the randomness of the process under investigation (by inferring the values of the Gamma shape parameter). At one extreme, with a Gamma shape value of 1, the process is memoryless exponential, where future events are not predictable by present events, and present events are not predictable by past events. At the other extreme, we have Gaussian-like processes, where present and past events can be used to predict future events. The continuous Gamma family spans all these different states and serves (in an MLE sense), in the human empirical biorhythmic data, as an accurate representation of the time series (random process) under consideration.

We then pair the Gamma NSR with the Gamma skewness, giving a picture of the tail of the distribution. This builds a parameter plane to represent the differentiation between the control case and the pain case for each of the four tasks under consideration. Figure 3K shows the parameter plane for the scalar Gamma NSR values obtained using the Euclidean norm of the Gamma NSR vector (as in Figure 3I) corresponding to the ophthalmic, maxillary, and mandibular regions of the face. The PDFs corresponding to each region and control *vs*. pain condition for this example are shown in Figure 3L.

### 2.5 Heart data MMS and gamma process analysis

The electrocardiographic (ECG) time series data registered by the MC10 Biostamp sensor, shown in Figure 2F and located on the chest area as depicted in Figure 2H (rightmost panel), was used to isolate the R-peaks and obtain the inter-beat interval (IBI) series. These are shown in Step 1 and Step 2 of Figure 4. Upon IBI acquisition, the series underwent frequency and time-domain analysis, also depicted as Step 3 and Step 4 of Figure 4, respectively. The power spectrum decomposition revealed the power of the signal across different frequency bands over time, dividing the frequency ranges into low- and high-frequency band regimes. These have been previously identified in the literature as sympathetic (low-frequency band spanning 0.04–0.15 Hz) and parasympathetic (high-frequency band spanning 0.15–0.40 Hz.) (Laborde et al., 2017; Vagedes et al., 2023). The former is associated with *fight-or-flight* states and the latter with rest-and-digest states (Vagedes et al., 2023).

A useful visualization method to show power across the frequency bands over time is the magnitude scalogram, shown in Figure 4 with the corresponding sympathetic *vs.* parasympathetic nervous system subdivisions. Lastly, the time domain analysis involves the Poincare plot, which examines the dispersion of the scatter formed by the RR times (in ms at time *t*) *vs.* the RR times advanced one frame (at time *t* + 1), fitted by an ellipse that identifies the principal axes of variance (standard deviations) as SD1 (shorter axis associated with the parasympathetic system) and SD2 (longer axis associated with the sympathetic system) (Hsu et al., 2012; Kamen et al., 1996). Motivated by prior results correlating the Gamma NSR with the SD2 values (Elsayed, 2024), we investigate whether the changes in SD2 and the changes in Gamma NSR of the heart IBI signal correspond in any way to the changes in SD2 and Gamma NSR of the face signals. In both cases, we use the MMS described in the previous section, adapted here to the IBI in Equation 3.


$$\mathrm{MM}S_{\mathrm{IBI}}=\frac{\mathrm{Pea}k_{\mathrm{IBI}}}{\mathrm{Pea}k_{\mathrm{IBI}}+\mathrm{Avr}g_{\min\;\text{to}\;\min}}$$ (3)

### 2.6 Statistical comparisons

To evaluate the statistical significance across Gamma distribution parameters in pairwise comparisons between conditions, we employed the non-parametric Wilcoxon rank-sum test. For more than two groups, we used MATLAB routines to implement the Kruskal–Wallis test, a nonparametric extension of the one-way ANOVA, which builds on the Wilcoxon rank-sum test. This test compares the medians of the groups of data in the parameter of interest to determine if the samples come from the same population. To compute the test statistics, the test uses the ranks of the data by sorting it from smallest to largest, rather than using the numeric values, and takes the numeric index of this order. If observations are tied, the rank is equal to the average rank of all tied observations. The *F*-statistic used in classical one-way ANOVA is replaced by a chi-square statistic, and the *p*-value measures the significance of the chi-square statistic. The Kruskal–Wallis test assumes that all samples come from populations with the same continuous distribution, apart from possibly different locations due to group effects, and that all observations are mutually independent. Both assumptions are satisfied in our data. In contrast, classical one-way ANOVA replaces the first assumption with the stronger assumption that the populations have normal distributions, which is violated in our empirical data.

We followed this with the MATLAB function multcompare to perform pairwise comparisons through an interactive graph that shows the significantly different means with disjoint (non-overlapping) intervals (Hochberg and Tamhane, 1987).

### 2.7 Correlations between heart and face gamma NSR

To explore the relationship between facial biorhythms and the heart’s IBI timings, we used the differences in Gamma scale (the Gamma NSR) from Pain relative to the control (no pain) condition of each task. Linear polynomial fitting *R*-square measurements and goodness-of-fit parameters are reported for each task across 21 participants for whom both heart and face data sets were available and collected simultaneously. We used the curve fitting toolbox of MATLAB with bicubic interpolation.

### 2.8 Transfer entropy measurements

We treat the two standardized micro-movements spikes (MMS) time series signals derived from the face and the heart’s IBI as random processes that are well characterized empirically by the continuous Gamma family of probability distributions. We empirically estimated these families as they changed for each participant from task to task in the control condition. We also estimated these families as they shifted from the control to the pain condition across all four tasks. We aimed to further understand the relationship between these two streams using the concept of transfer entropy (TE).

Given process *X* (representing the face MMS) and process *Y* (representing the heart’s IBI MMS), TE quantifies the amount of uncertainty reduced in future values of *Y* by knowing the past values of *X* given the past values of *Y*. If *X_t_* and *Y_t_* for *t* ∈ ℕ are two random processes, and the amount of information is measured as Shannon’s entropy (Equation 4).


$$H(X)=\sum_{x\in X}p(x)\log p(x)$$ (4)

where Σ denotes the sum over the possible values of the variable *x* and we adopt base 2 (unit of bits) to measure the information of the variable, the conditional mutual information with the history of the influenced variable in the condition is denoted as (Equation 5):


$$T_{X\to Y}=I(Y_{t};|X_{t-1:t-L};|Y_{t-1:t-L})$$ (5)

And the TE is denoted as (Equation 6):


$$T_{X\to Y}=H(Y_{t}|Y_{t-1:t-L})-H(Y_{t}|,Y_{t-1:t-L}|,X_{t-1:t-L})$$ (6)

We used the Information Dynamics Toolkit (JIDT)^1^ infodynamics-dist-1.6 toolbox in MATLAB with an open-source Java implementation by Joseph T. Lizier, Ipek Özdemir, Pedro Mediano, Emanuele Crosato, Sooraj Sekhar, Oscar Huaigu Xu, and David Shorten. We used the continuous-valued TE functions in both directions, setting *X* in one direction as the face MMS and *Y* as the heart IBI MMS, and in the opposite direction, setting *X* as the heart IBI MMS and *Y* as the face MMS.

We asked if knowing the face MMS and the heart IBI MMS from the past could reduce the uncertainty of the present face MMS. We also reversed the question and asked if the current heart IBI MMS uncertainty decreased given the past face and heart IBI MMS. To that end, we used windows of 50 frames with 10% time into the past (5 frames or ~166.6 ms at 30 Hz video resolution) for 300 frames representing 10 s of data from the face or heart IBI states. We note here that despite the disparate sampling resolutions between the video capture and the Biostamp MC10 sensor, the standardized MMS data type brings the two derived signals onto a comparable scale of peaks relative to the empirically estimated mean. We can therefore investigate this TE question.

For each task, we sorted the TE values across the 21 participants for the TE obtained in one direction $T_{X\to Y}$ and plotted the TE in the other direction $T_{Y\to X}$ , for each participant. This helped us appreciate the deviations of one curve from the other in those participants where the TE was low *vs.* the cases where the TE was high, relative to the curve of individually sorted values.

$T_{\mathrm{IBI}\;\text{Heart}\to \text{Face}}$ was used as the sorted participants in one comparison to examine $T_{\text{Face}\to \mathrm{IBI}\;\text{Heart}}$ in both the control condition and the pain condition.

Then, the sorted participants $T_{\text{Face}\to \mathrm{IBI}\;\text{Heart}}$ were used to plot the $T_{\mathrm{IBI}\;\text{Heart}\to \text{Face}}$ values in both conditions.

Lastly, we took the differences in each case, relative to the sorted data, and for each task examined the differences in TE in both directions. We note here that our goal was more modest than establishing causality. We merely ask whether knowledge of the recent past in both signals lowers the uncertainty of one of the signals (the face or the heart, by comparing them one at a time). Since we are measuring this based on the shifts in Gamma NSR and have established the empirical relationship between the Gamma NSR and the distribution shape, we know that decreasing the NSR parallels increasing the shape values away from the memoryless Exponential ranges of the Gamma family. We also know that in other motor biorhythms, this is described by a scaling power law with an inverse relation between the Gamma NSR and the Gamma shape—as the NSR decreases (indicating a higher signal-to-noise ratio), the shape shifts to more symmetric regimes, featuring more predictive signals and therefore lowering the uncertainty of the system (Torres et al., 2013). The use of TE here on the standardized MMS streams of the face and the heart is justified by the Gamma NSR serving as a proxy for the full distribution, a relationship that we have uncovered empirically.

## 3 Results

### 3.1 Tight linear relation (scaling power law) describes the log gamma shape and log scale parameter’s fit of the IBI MMS peaks

**Figure 5:**
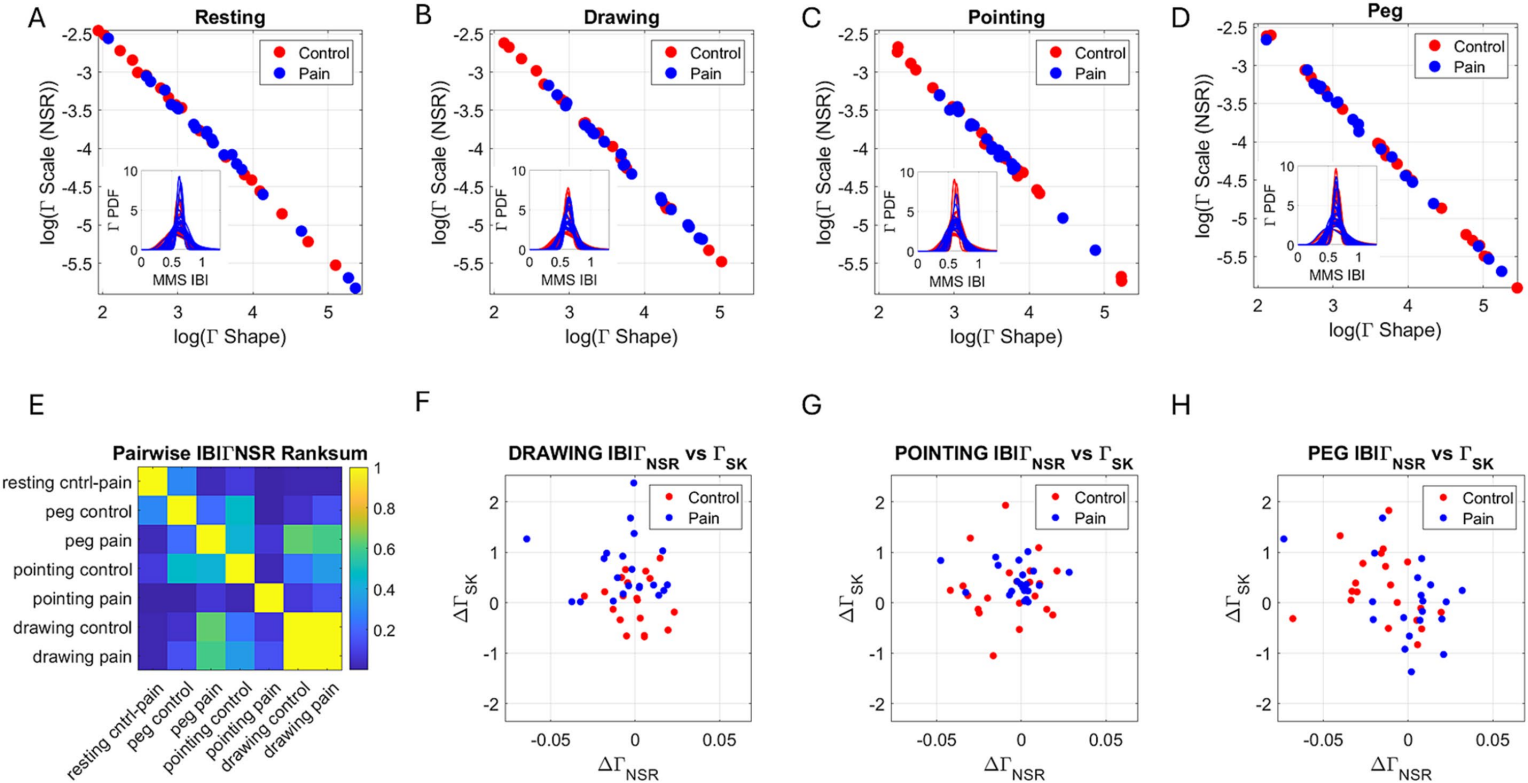
The continuous gamma family of probability distributions fits the empirical IBI data well in a maximum likelihood estimation sense, with 95% confidence. **(A)** Control and pain conditions at rest, **(B)** Drawing, **(C)** Pointing, and **(D)** Peg tasks. Each (Г_shape_, Г_scale_) point on the gamma parameter plane is plotted to reveal a self-emerging pattern; the log–log scatter aligns according to a tight linear negative correlation: as the distribution becomes more symmetric (with increasing shape values), scale (dispersion) values decrease. The scale is the noise-to-signal ratio (NSR, variance over the mean). **(E)** The pairwise comparison of the NSR according to the rank-sum test reveals statistically significant differences (*p* < 0.01) for all tasks during the pain state, relative to the absolute difference between pain and control states for the resting task. **(F–H)** The parameter plane represents the difference in NSR vs. the difference in skewness for each task relative to the resting task, in both the control (red) and pain (blue) blocks.

The frequency histograms of the MMS time series derived from the IBI were well fit by the continuous Gamma family of probability distributions (PDF), which generate a unique family with personalized shifting patterns for each participant. At the cohort level, as with other biorhythms, the scatter of points representing the (Г_SHAPE_, Г_SCALE_) values, whereby each point corresponds to a participant’s signature, also followed a tight linear relationship on the log–log Gamma parameter plane. In this sense, as in other biorhythms well characterized by the MMS Gamma process, knowing one parameter enables us to infer the other. This can be appreciated in Figures 5A–D for each of the tasks and for both the control (red dots) and the pain (blue dots) conditions. The insets of the figure reflect the empirical families of Gamma PDFs derived from the empirically estimated Gamma shape and scale parameters, with 95% confidence. Supplementary Table 1 shows the fitting values, including the slope and intercept values, as well as the centered data mean and standard deviation values for each task (Resting, Drawing, Pointing, and Peg) and condition (Control *vs*. Pain).

As the shape parameter increases from the limiting value of 1 (the memoryless exponential regime) toward higher values characteristic of heavy-tailed distributions and even higher values seen in symmetric Gaussian distributions, we observe a decrease in scale (dispersion) values. The scale represents the dispersion (spread) and is also the NSR, as explained in the Methods. The negative correlation shown by the tight linear fits to the log–log values implies that as noise decreases, the process becomes more predictable, while an increase in noise leads to a more random process (approaching the memoryless exponential regime). Pairwise comparisons of the NSR parameters across participants revealed significant differences (Wilcoxon rank-sum test *p* < 0.01) when comparing resting in control *vs*. resting in pain. This pattern also held for the other tasks when examining this relative difference (except for the peg task during the control block). This can be seen in the first row and first column of Figure 5E, which depicts the color-coded matrix of probability values from the rank-sum test. As participants performed different tasks, their individualized families of distributions changed in shape and scale parameter values, demonstrating the non-stationary nature of heart IBI timings. The shifts from control to pain also highlight the susceptibility of this parameter to pain and suggest it serves as a good proxy for states of pain.

### 3.2 Heart IBI gamma PDF skewness and NSR shift across tasks relative to resting state

Given that knowing the Gamma NSR allows us to accurately infer the Gamma shape parameter, we turned our attention to the Gamma scale parameter as a proxy for the Gamma PDF spanned by each personalized family (for each participant) and examined it in relation to the corresponding Gamma skewness. To that end, we built a parameter plane whereby points represent the (Г_NSR_, Г_SKEWNESS_) values. The scale indicates the NSR (Gamma variance divided by Gamma mean) and provides information on the predictability of heart IBI timing, while the skewness provides information on the tail of the distribution. At 0 skewness, the distributions are symmetric. Positive values indicate an accumulation of larger MMS (standardized deviations from the Gamma mean for the heart IBI), suggesting shorter interval times between R-peaks, which indicates an average faster heart rate and respiration rhythms. Negative values indicate an accumulation of smaller MMS, suggesting longer interval times between R-peaks, which indicates an average slower heart rate and respiration rhythms. This occurs due to the standardization of the heart IBI series by Equation 1. In Equation 1, the term in the denominator indicates the local averages of the signal comprised of the points from local minimum to local minimum surrounding the local peak (maximum). If this value is on average small, the numerator is larger. Thus, shorter IBI timings on average (faster heart rate and respiration rhythms) will produce larger MMS deviations from moment to moment.

For each task, we obtained the differences in NSR and skewness relative to the resting task and noted differences in shifting patterns for each task. During the control block, the Drawing Task exhibited a decrease in skewness values that contrasted with the Peg Task, where most participants showed an increase in skewness. For the positively skewed Gamma PDF family, a negative shift indicates that the tail shrinks (fewer large MMS fluctuations or, equivalently, larger IBI averages impacting the scaling in Equation 1). On the other hand, a positive shift indicates a heavier right-tail, with larger MMS fluctuations contributing to this. Given the computation of standardized MMS, i.e., with the denominator containing the average IBI values between the IBI local minima surrounding the IBI maximum, these positive shifts with an increase in MMS then show lower heart IBI timing values on average. As explained, lower heart IBI timing values often arise from faster heart rates, which are evident in anxious, stressful states. In this sense, drawing and pointing increase the skewness values (Figures 5F,G, respectively), while the Peg Task includes some participants who increase and others who decrease the skewness during the pain block, relative to the control block (Figure 5H).

The Gamma NSR also shifts in each task relative to rest. In the control block, the drawing task shows a balanced shift, with some participants decreasing and others increasing the NSR relative to the resting task for both the control and pain conditions. During the Pointing Task, the control case exhibits a greater spread than during pain, with the scatter biased toward the negative shift. Here, negative means that the NSR decreased during the drawing task in both the control and pain blocks, compared to the resting task. Lastly, the Peg Task shows a negative shift during the control block but a positive shift during the pain block. This indicates that, relative to the Resting Task, the Peg Task evokes higher variability in the IBI fluctuations for most participants. Given the relationship in Figures 5A–D between the Gamma NSR and the Gamma shape, and considering that higher noise leads to more randomness in the process, it is reasonable to infer that, as in the Drawing Task, most participants in the Peg Task exhibit more erratic heart rate patterns during the pain state than during the control state. Interestingly, unlike pointing, which is mediated by kinesthetic (speed and proprioceptive posture feedback), the Drawing Task and the Peg Task are more cognitively demanding. In addition to the speed and posture feedback during the transport phases of the hand motions, these two tasks are also influenced by pressure (haptic) feedback. Consequently, heart IBI captures the increasing difficulties of the tasks: biomechanical goals (Pointing Task), biomechanical and haptic goals (Peg Task), and those plus cognitive/memory goals (Drawing Task).

### 3.3 Tight linear relation (scaling power law) describes the log gamma shape and log scale parameters fit of the facial speed MMS (micro-fluctuations)

**Figure 6:**
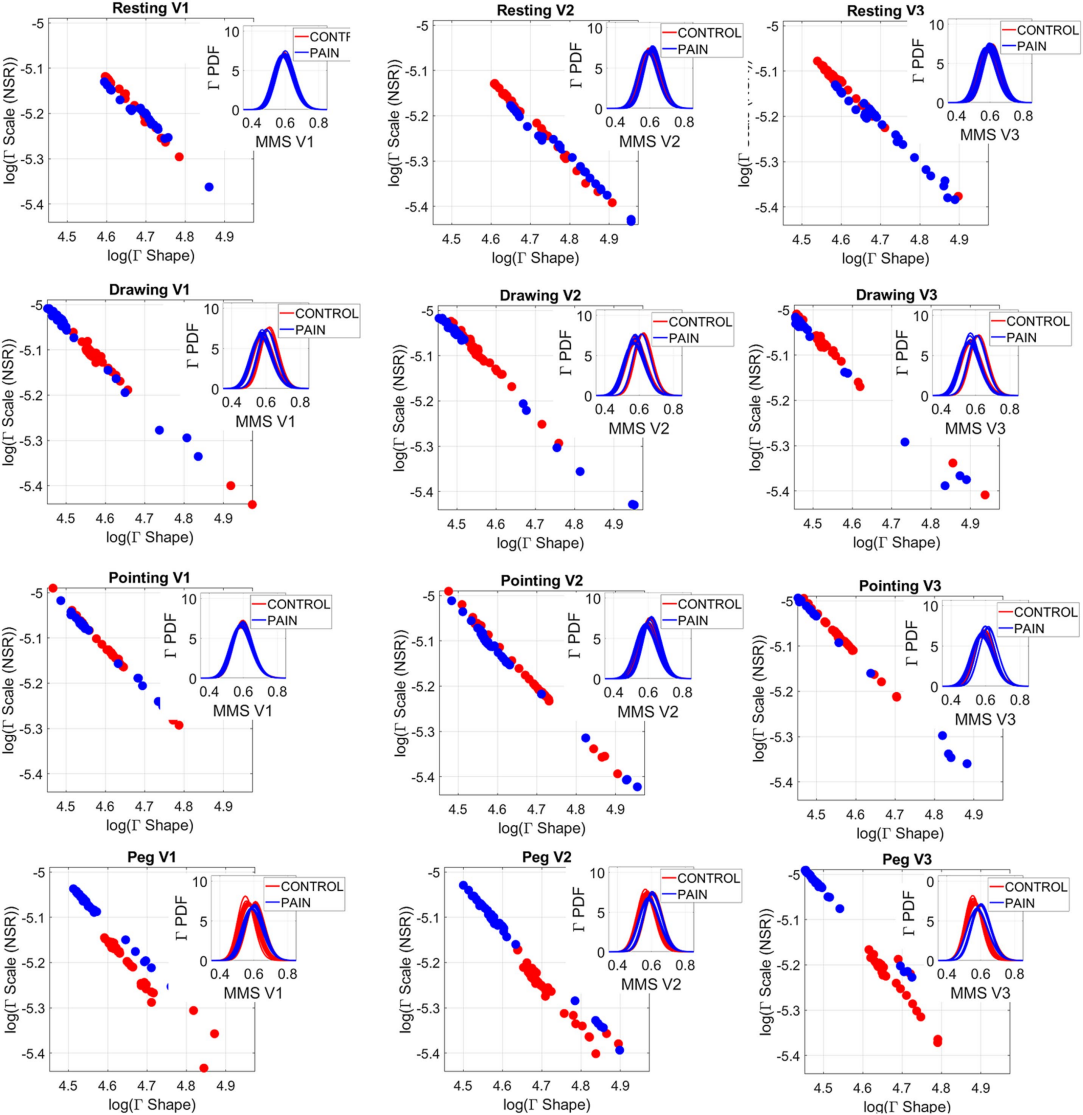
Gamma parameters for facial micro-movements across all regions V1, V2, V3 for all 4 tasks (resting, drawing, pointing, and peg) and for control (red dots) and pain (blue dots). Each dot represents a participant’s empirically estimated (Γ_shape_, Γ_scale_) measurement, spanning a family of PDFs for each participant. Insets show the gamma PDFs from the empirically estimated shape and scale parameters. Scatters are well fit by exponential curves, and as in the heart IBI case of Figure 5, the log–log representation is well fit by a tight linear negative correlation whereby as the gamma NSR decreases, the gamma shape increases (see Supplementary Table 2).

As we focus on the Gamma NSR, the parameter space spanned by the values along each region forms a three-dimensional scatter that we can also examine across participants for each of the control and pain conditions and across all four tasks. Figure 6 shows the scaling power law relations between the log Gamma NSR and the log Gamma Shape. These comparisons highlight an increase in noise levels across all three regions of the face during pain in the Pointing, Peg, and Drawing Tasks, relative to the control condition. Not only do we observe a shift in noise patterns in the ophthalmic V1 area, but we also see this shift in the maxillary V2 and mandibular V3 areas, where the noise and randomness of the moment-by-moment micro-fluctuations in speed deviations from the empirical mean (as measured by the standardized MMS) increased significantly, particularly for the Peg Task. Supplementary Table 2 presents the linear polynomial fits and goodness of fit parameter values, which were obtained using the Curve Fitting Toolbox of MATLAB R2023b.

### 3.4 Facial V1 micro-movements shift across tasks during control state

**Figure 7:**
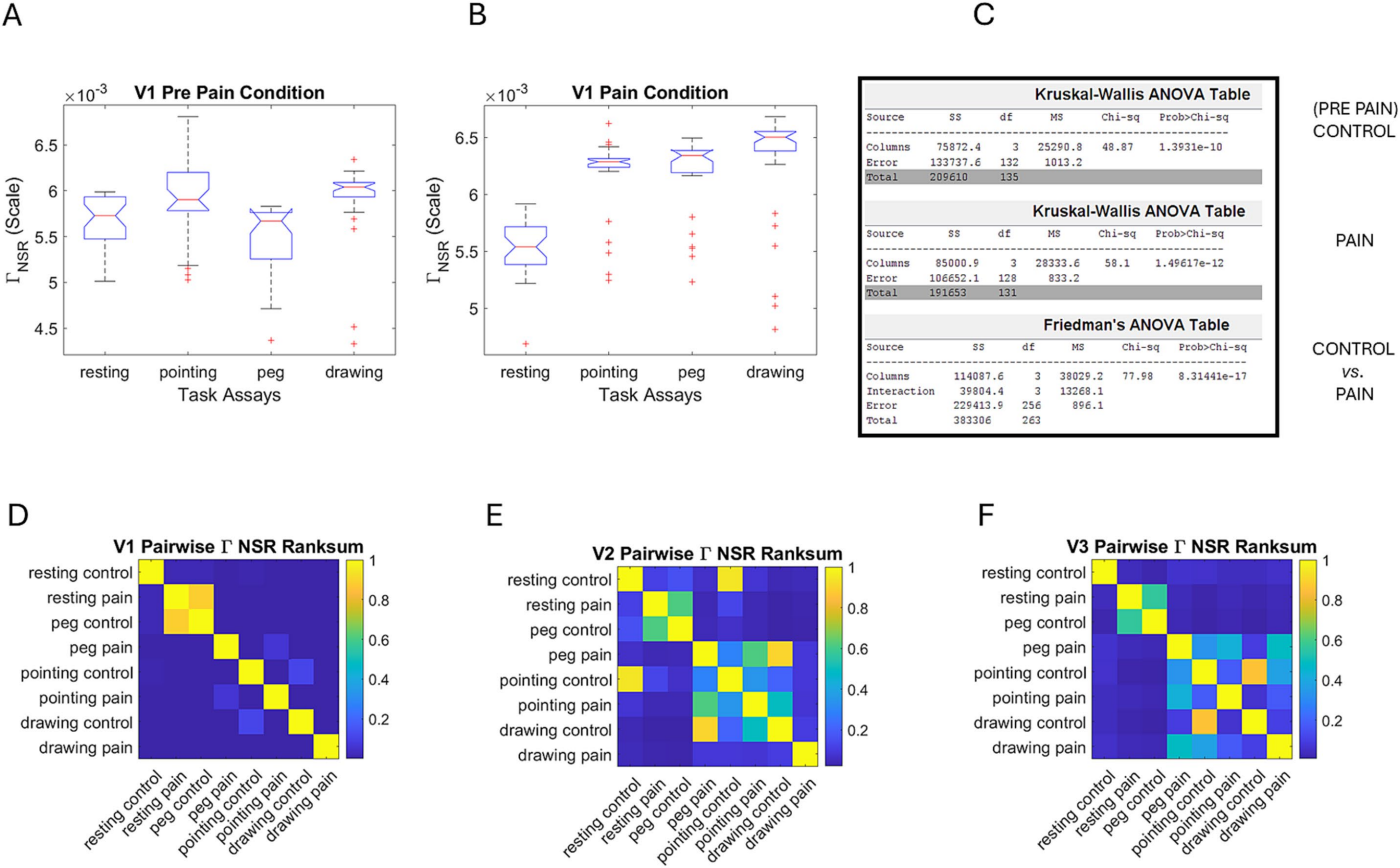
Statistical tests of the Gamma parameters for facial micro-movements. **(A)** Control condition (pre-pain) shows statistically significant differences in the Gamma NSR parameter according to the non-parametric Kruskal–Wallis test for the ophthalmic area V1 across all tasks. The MATLAB Multcompare test reveals *p* < 0.01 for all pairwise comparisons relative to the resting task. **(B)** During the pain condition, these differences are accentuated. **(C)** Stats table for comparisons A and B and for the control *vs*. pain comparison according to the Friedman’s test. All comparisons yield *p* < 10^−10^. **(D)** Pairwise comparisons of NSR rank-sum test in ophthalmic area V1, **(E)** maxillary area V2 and **(F)** mandibular area V3.

The V1 (ophthalmic) region of the face, comprising the eyebrows, the eyes, and part of the nose, revealed significant differences in the Gamma NSR signatures across tasks, according to the non-parametric Kruskal–Wallis statistical test. This is shown for the control (pre-pain) state in Figure 7A across tasks. Figure 7B shows the within comparisons for the pain condition. The tables in the figure (output by MATLAB) summarize the results in Figure 7C, where we also show the output of the Friedman’s test comparison between the control and pain conditions. Figure 7D shows the pairwise *p*-values obtained from the rank-sum test on the Gamma NSR parameters (equivalently the negatively correlated Gamma shape parameter) across all tasks and conditions for region V1. Figures 7E,F show the pairwise comparisons of the empirically estimated Gamma NSR parameters for V2 and V3, respectively. As can be seen, V1 exhibited significantly different families of Gamma PDFs across tasks, while V2 and V3 subregions only show such differences in relation to the resting task. Given the marked shifts in Gamma PDFs for V1, we next focus on this facial subregion to illustrate the comparisons and examine its relationship with the heart IBI streams.

### 3.5 V1 micro-movements shift across tasks during pain states

As with the MMS streams derived from the heart IBI timings, here we derived the MMS from the V1, V2, and V3 regions of the face and found the best fit for the MMS micro-peaks using the continuous Gamma family of PDFs in an MLE sense. We empirically estimated the shape and scale parameters with 95% confidence. As we examined the shape and scale relations in Figure 6, we observed a shift in the Gamma NSR from the control to the pain states. This shift is most pronounced for the Peg Task and the Drawing Task but is also present in the biomechanical Pointing Task. The insets across the panels show the shift in the Gamma PDFs derived from the empirically estimated parameters. Notably, there is an upward shift with the pain condition within the Pointing, Peg, and Drawing Tasks across all facial subregions. This increase in noise is also accompanied by a decrease in the shape value, tending toward the memoryless exponential range of the Gamma family (approaching the extreme value of 1 that denotes randomness). This pattern is similar to those observed in the IBI signal.

**Figure 8:**
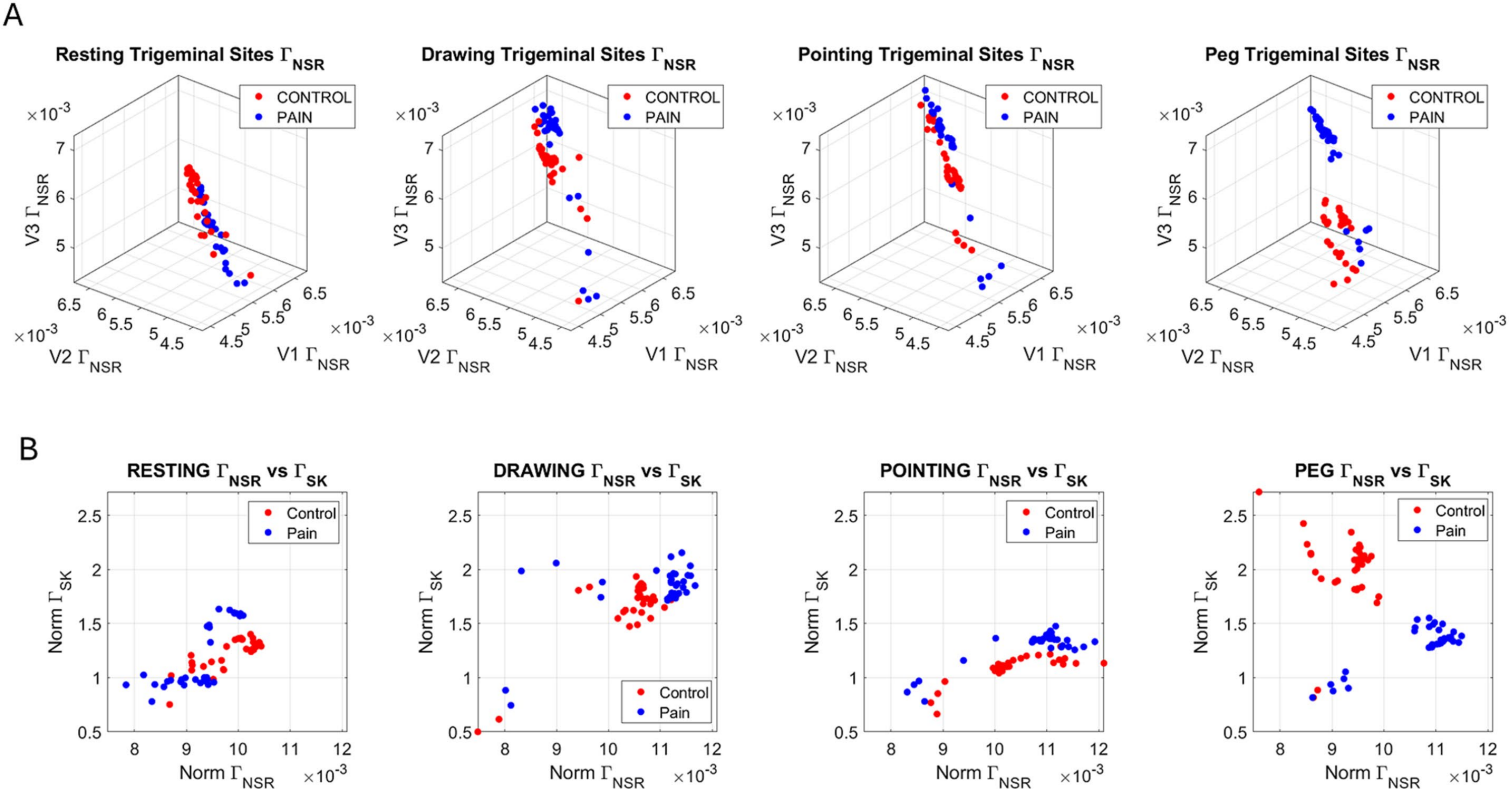
Different representations of the face data capture shifts in gamma families of probability distributions from control to pain conditions and across the different tasks. **(A)** Parameter space spanned by V1, V2, V3 gamma NSR with control (red dots) and pain (blue dots), where each dot represents a participant, and each parameter space denotes the state of the face during the task. **(B)** The Euclidean norm of the parameters vector (V1, V2, V3) is obtained for both the NSR and the skewness, with the scalar quantities represented on a parameter plane spanned by the norm Г_NSR_ and the Г_Skewness_. Notice the different shifting patterns (see details in the results section).

Figure 8A shows the shifts of the Gamma NSR across the V1, V2, and V3 regions represented as points in the 3D parameter space spanned by these triads of values. The scatter shows the increases in noise across the face, while Figure 8B summarizes these shifts within the parameter plane defined by the Euclidean norm of the (V1, V2, V3) points taken as vectors from the (0, 0, 0) origin. These are expressed as the scalar magnitudes of such vectors for both the NSR and skewness components across the face. During the Resting Task, we observe two groups of participants within the pain case: one group shows increasing skewness relative to the control case, while the other either decreases it or maintains it at the lower end of the control values.

An increase in skewness indicates a heavier tail of the PDF, suggesting larger values of the MMS. Recalling Equation 1, the MMS are absolute deviations from the empirically estimated Gamma mean speed amplitude. Larger values of such normalized deviations from the empirical mean occur when the average speed (in the denominator term of the normalizing expression) is smaller, i.e., slower speed. In contrast, lower skewness toward a more symmetric density corresponds to lower MMS values when the average speed is higher. While pointing in pain, the skewness increases (indicating a lower average speed of the facial MMS micro-fluctuations). In contrast, skewness decreases during the Peg Task in pain (denoting a higher average speed of the facial MMS micro-fluctuations), and noise increases (which also introduces more randomness and decreases shape values). The Drawing Task in pain also increases noise and randomness levels, while skewness remains comparable to that of the control condition for most participants. These results demonstrate very different patterns for the Peg Task and the Drawing Task, which involve haptic feedback and a higher cognitive and memory load compared to pointing at a visual target and receiving feedback from hand speed and the rotational arm joints as the transport phase of the motion unfolds. In all three tasks, pain levels increased relative to rest, but the speed of the facial micro-movements behaved differently.

### 3.6 Distribution parameters of V1 region distinguish tasks and conditions

**Figure 9:**
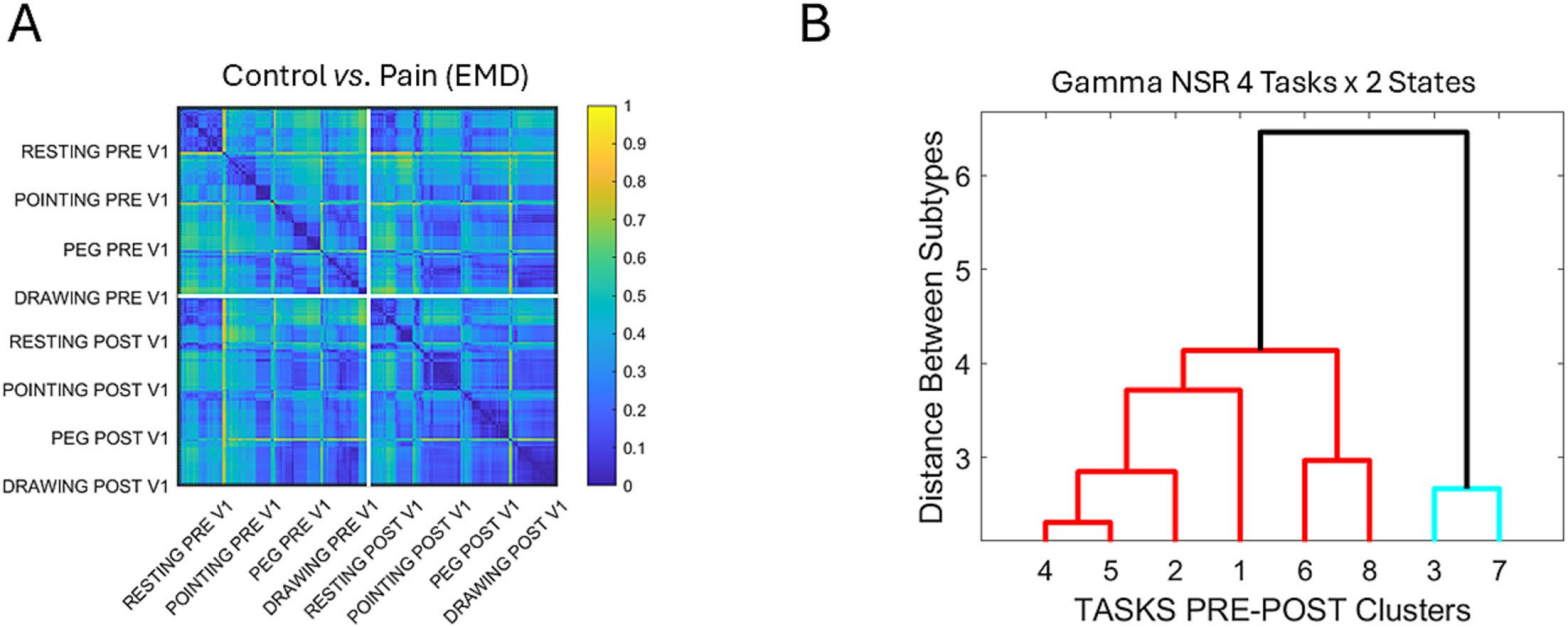
Individualized probability distribution families across participants are compared pairwise using the EMD and grouped into self-emerging clusters. **(A)** Matrix of EMD values denoting the normalized distance (color bar) for each participant in the V1 area (the most significantly impacted by tasks and pain) – PRE refers to the control (before pain condition) and POST refers to the pain condition. **(B)** Tree clustering of the participants into 8 groups according to the EMD values in **(A)**, obtained from the distributions of gamma NSR parameters empirically estimated. Cluster composition of control *vs*. pain PDF percentages is detailed in the main text.

The statistical significance of the pairwise comparisons across the tasks and conditions for the ophthalmic region V1 discussed in Figures 7A–D motivated us to further compare the PDF families in this region. To that end, we used the earth mover’s distance (EMD) (Monge, 1781; Stolfi and Guibas, 2000) to compare pairwise, for each participant, the PDFs generated by the MMS derived from the speed peaks. The results are shown in Figure 9A, distinguishing the pre-pain and during-pain (denoted post-pain) activities for each task. This information was fed into a clustering tree, resulting in the identification of 8 subgroups in Figure 9B. Their composition from left to right was 96% control, 57.1% control, 52.3% control, 100% control, 50% pain, 64.2% pain, 100% control, and 100% control.

### 3.7 Linear trend between NSR shifts of face and heart IBI control vs. pain

**Figure 10:**
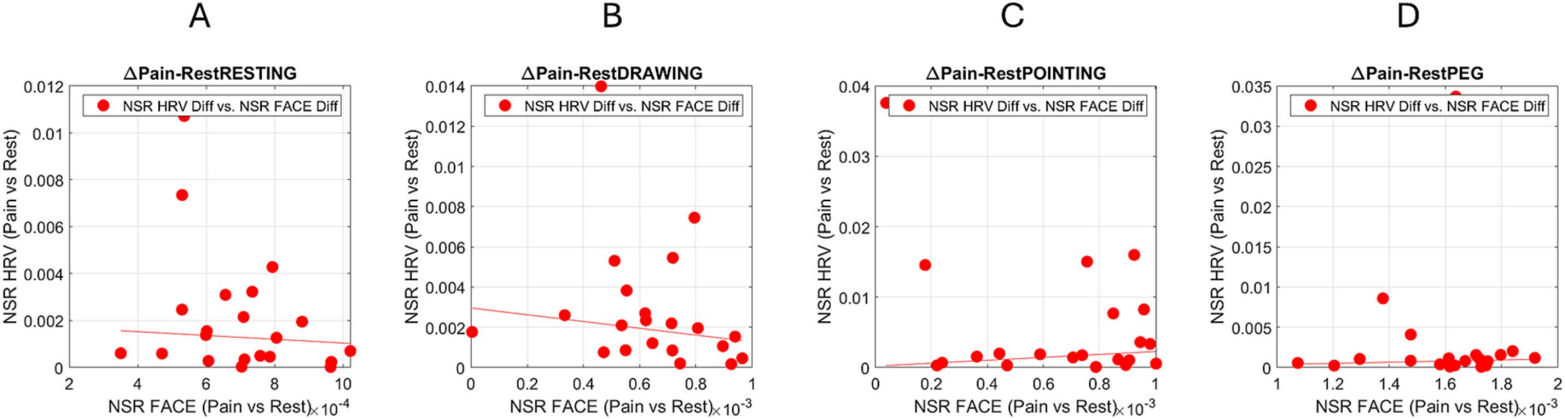
Linear correlations between the difference in gamma NSR shifting from control resting to the pain condition for each task. **(A)** Resting task. **(B)** Drawing task. **(C)** Pointing task. **(D)** Peg task. Supplementary Table 3 reports the correlation values and the goodness of fit.

The differences between pain and control Gamma NSR were obtained from facial and heart IBI MMS. Linear polynomial fits were obtained for the scatter of each task, revealing a linear trend for the Pointing Task, the Peg Task, and the Drawing Task. These are reported in Supplementary Table 3 and shown in Figure 10.

### 3.8 Transfer entropy: the face reflects the increasingly erratic heart signal with pain

Given the linear correlation between the HRV rates of change of noise and that of the ophthalmic facial region V1, we obtained TE values for these time series. The TE values for $T_{\mathrm{IBI}\to \text{Face}}$ were sorted in increasing order across participants, i.e., for the case when knowing the past heart IBI & face signal reduced the uncertainty of predicting the face signal. For each participant then, the TE in the opposite direction, i.e., when knowing the past heart IBI & face activity reduces the uncertainty of predicting the heart IBI activity, $T_{\text{Face}\to \mathrm{IBI}}$ was plotted such that we could see for each participant, the deviation of this TE value relative to the sorted (from low to high) TE curve.

**Figure 11:**
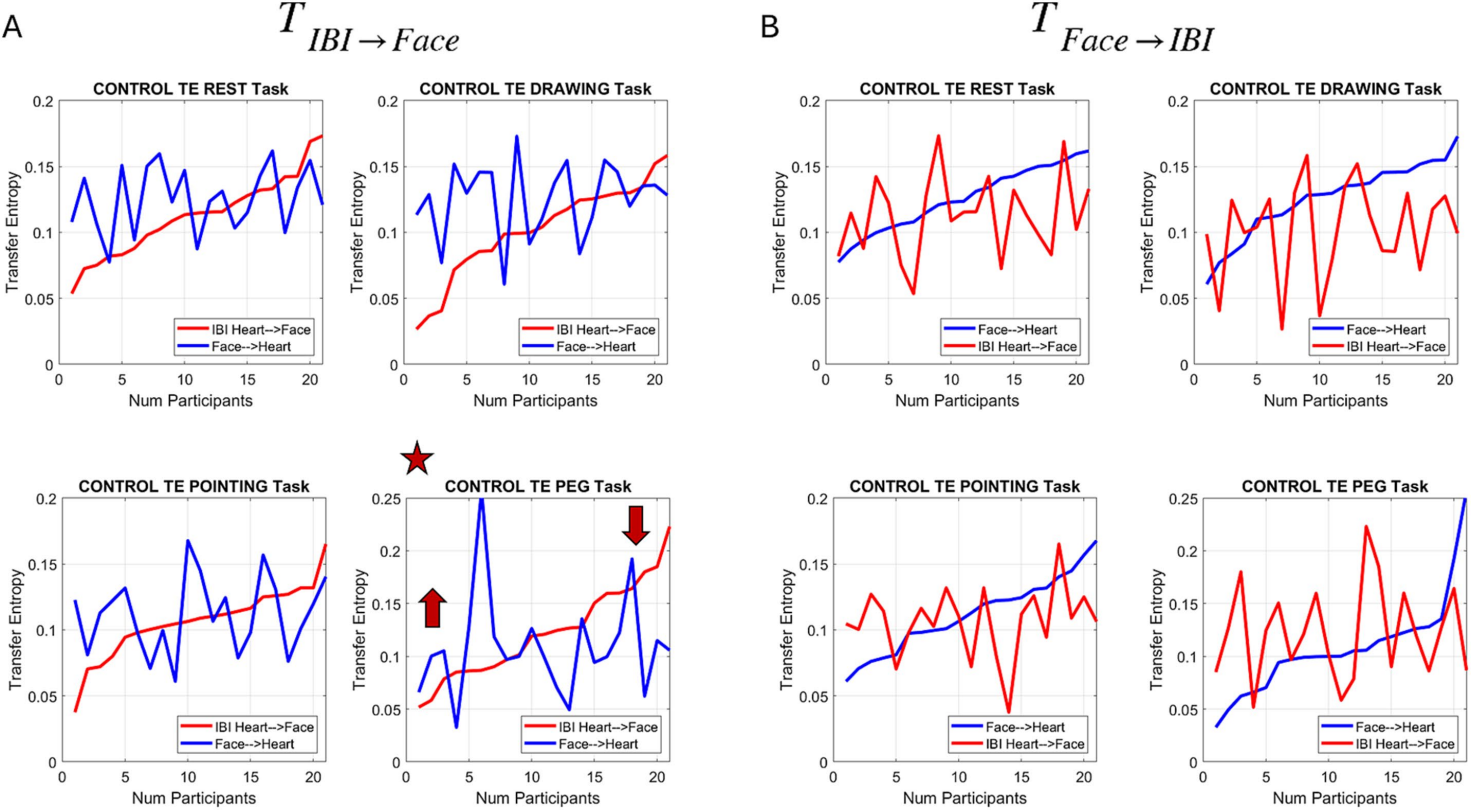
Measures of transfer entropy during the control session for all 4 tasks and across participants. **(A)** TE sorted in increasing levels within the sample of participants (used as an individualized threshold) for the case where the past heart IBI & face activity predicts the current face activity. Superimposed for each participant is the TE curve (denoted face → IBI) where past heart IBI & face activity predict current heart-IBI activity. The plot with the star highlights the peg task where participants with lower values of TE (in IBI → face) express higher TE (in face → IBI) for lower thresholds and lower TE (in face → IBI) for higher thresholds (of IBI → face TE). **(B)** Same analysis as in **(A)** but inverting the process by sorting the face → IBI TE signal across participants.

The resulting curve for the control case (no pain) revealed that in the peg task, during this control condition, there seemed to be an individualized threshold such that those participants with a lower value of TE in the case of $T_{\mathrm{IBI}\to \text{Face}}$ (red curve) tended to have more pronounced effects predicting the heart IBI signal. Figure 11A shows the traces for all tasks and points out the Peg Task case. Figure 11B shows that this trend is not present when we invert the comparison.

Interestingly, the Peg Task was the one with the highest linear correlation (adjusted *R*-square = 0.84 in Supplementary Table 3 and Figure 10) between the face and heart signals, capturing the shifts in Gamma NSR from control to pain. This motivated us to perform this analysis for all the tasks during the pain condition. We sought to determine the extent to which the pattern observed in the Peg Task would also be present in the other tasks when participants experienced pressure pain.

**Figure 12:**
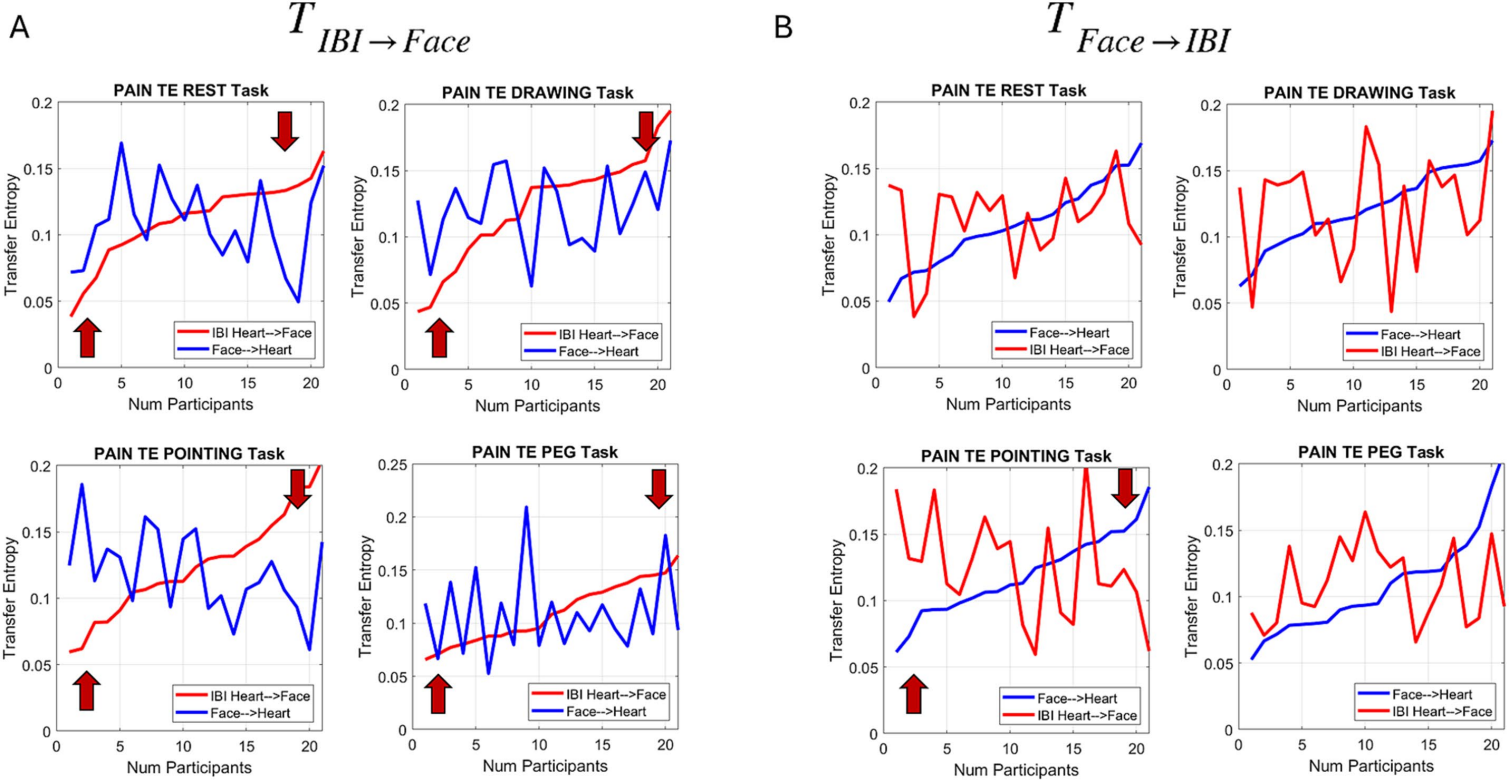
Measures of transfer entropy during the pain session for all 4 tasks and across participants. **(A)** Identified pattern in the Peg Task is consistently observed during the pain state in other tasks. **(B)** Results from the reverse analysis also show marked differences from the control case (peg task highlighted for comparison purposes).

Figure 12A reveals that during the pain conditions, the effects observed in the control condition for the peg task extend across all 4 tasks. There seems to be an individualized threshold whereby individuals with lower TE (more uncertainty), in the case where past activity of the heart IBI and FACE combined is predictive of present face activity, tend to have higher TE values. Inverting this analysis also shows marked changes relative to the control condition (Figure 12B, where we highlight the peg condition for comparison purposes).

In this random draw of the population, two subgroups emerge where about half of the participants expressed higher TE levels of $T_{\text{Face}\to \mathrm{IBI}}$ when the $T_{\mathrm{IBI}\to \text{Face}}$ was lower and the other half show the opposite trend. This was the case even at rest, while experiencing pain.

**Figure 13:**
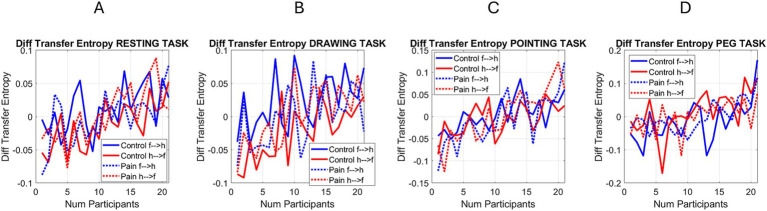
Differences between TE for each of the control and pain conditions across participants (f → h for face to heart and h → f for heart to face). **(A)** Resting task. **(B)** Drawing task. **(C)** Pointing task. **(D)** Peg task.

Given the patterns during the pain condition, we then proceeded to obtain the relative differences between the sorted values (smoothly increasing curve) and the varying one, during both the control and pain cases. The results are presented in Figure 13 in the order in which the experiments took place. We appreciate the overall decrease in TE relative differences during the pain condition (dotted lines), both when the past face and heart IBI signals together better predict the present heart signal and when they better predict the present face signal. In both comparisons, the Resting Task and the Drawing Task show a higher difference in TE when the uncertainty in predicting the present IBI signal decreases, whereas the difference is not as pronounced for the Pointing Task and the Peg Task. Perhaps some adaptation or fatigue effect is responsible for this pattern.

Regardless of potential effects of fatigue or adaptation to pain, the face and heart activities seem not only correlated but also take turns leading and lagging each other in ways that knowing one in the recent past contributes to reducing the uncertainty of predicting the other in the present. The heart signals of individuals with greater uncertainty in past heart IBI (more erratic heart timing) increasingly allow face signals to show this dysregulation with higher certainty, a trend that fades away when the uncertainty of the past heart signal in predicting the face decreases.

A more regulated heart in the recent past does not contribute as much as a dysregulated heart to the prediction of present face signals. During the pain state, when the heart signal is more dysregulated, the recent past activity of the heart may be reflected in present facial micro-movement spikes, suggesting the face acts as a proxy for recent heart dysregulation. We sampled video of the face with a regular webcam at 30 Hz, ~every 33.3 ms, such that in our analysis, 5 frames represent approximately 166.7 ms of past heart activity considered to obtain the TE measure indicating a reduction in the uncertainty of the face activity. We will next discuss the implications of the present results, potential caveats of our study, and future steps in the research.

## 4 Discussion

This work examined the relationship between heart IBI signals and face micro-movement spikes, derived from 10-s-long videos and concurrently recorded in a subset of participants while they engaged in four tasks during a control condition (pre-pain) and during a pain condition (induced pressure pain). We leveraged open access software, the Open Face suite, to acquire our data from video (Baltrušaitis et al., 2016). These methods provide a grid of points that describe changes in pixel positions over time. By extracting the rate of change of position per unit time, we obtained the speed temporal profiles of 68 points across the grid and studied the ophthalmic (V1), maxillary (V2), and mandibular (V3) subregions of the face in correspondence with the biorhythmic data of heart IBI timings.

An empirical characterization of the standardized data type created in our lab, termed micro-movement spikes (MMS, or micro-peaks), was used to represent the moment-to-moment micro-fluctuations of both heart IBI peaks and face speed peaks, relative to their corresponding (empirically estimated) Gamma mean. In both time series, the biorhythmic micro-fluctuations output from the peripheral and autonomic nervous systems were well characterized by the (empirically estimated) continuous family of Gamma probability distributions. The Gamma shape and scale parameters provided a good representation, in an MLE sense, of the peaks of the standardized MMS data. Increases in the Gamma NSR (the scale parameter) corresponded to greater randomness in the micro-fluctuations, i.e., a decrease in the shape parameter toward the memoryless exponential regime of the Gamma family, where the shape is 1. This relationship was well fit by tight linear polynomial equations on the log–log shape-scale Gamma parameter plane (reported in Supplementary Table 1 for the heart IBI MMS and in Supplementary Table 2 for the face MMS data). Since this tight fit allows accurate inference of the Gamma shape from the Gamma NSR, we focused our analysis on a single parameter, the Gamma NSR, which is the Gamma scale parameter indicating the spread of the distributions. Each participant spanned a family of Gamma PDFs across the tasks (resting, drawing, pointing, and the grooved peg) and conditions (pre-pain or control and pain). We then used the Gamma NSR parameter in combination with another parameter, the Gamma skewness, to interpret our data.

Furthermore, given this power law description of the parameters of the distribution families that each participant spanned across tasks and conditions, we asked whether the two Gamma NSR parameters from the micro-fluctuations of the heart and face streams were correlated. First, we explored the activities of the three regions of the face and found that the ophthalmic V1 region maximally expressed the shifts in noise signatures from control to pain across the tasks. We then focused on this V1 region and found statistical significance for these effects.

The shifts in Gamma NSR of the face V1 region during the pain condition, relative to the control state, were tested against the shifts in Gamma NSR of the heart IBI. We found linear correlations with *R*-squares of 0.84 and 0.85 in the Pointing and Peg tasks, respectively (the last two tasks that the participants performed). One aspect of the pain assay may be adaptation, while another aspect of the tasks performed might be fatigue. The fact that the statistical effects were strong at the end of the peg block and that the correlation between heart and face was stronger in the Peg Task than during the Resting Task or the earlier Drawing Task suggests that pain adaptation or fatigue alone could not account for the pain effects quantified here.

To further explore the nature of the relationship between the heart and face signals, we calculated the Transfer Entropy between the two processes in both directions. This analysis identified an interesting pattern in the Peg Task during the control condition. By sorting the TE values (past activity of the heart and face decreasing the uncertainty of predicting the present face activity), we observed that the TE behaved in a notable fashion. Those participants with increasingly erratic heart activity (higher noise and randomness in the micro-peak fluctuations around the empirically estimated Gamma mean) expressed higher TE for predicting present facial ophthalmic activity. This was specific to the Peg Task in the pre-pain control condition. How would this finding reflect in other tasks with the Pain condition?

Using the same analysis during the pain condition extended the results to all other tasks. This finding suggested a sort of individualized threshold of TE indicating under what conditions the face V1 activity is predictive of heart IBI activity. It appears that face activity in the ophthalmic region can act as a proxy for predicting increasingly erratic heart activity. Intuitively, as pain escalates and the heart reveals it, the face can broadcast it in the ophthalmic region around the eyes. Here, we could quantify this using the moment-by-moment fluctuations of the standardized MMS (micro peaks) of both the heart IBI and the face.

We performed the comparison in the opposite direction and observed congruent results. This motivated us to obtain the absolute differences between the sorted (monotonically increasing) TE values in one direction and the changing TE curve in the other direction. We found that the control condition exhibited wider ranges of differentiation between the direction of comparison, with the signal predicting the face from the dysregulated heart leading in the resting and drawing tasks but mostly lagging in the pointing and peg tasks. In contrast, the pain condition showed narrower ranges of differentiation across tasks. This suggests that during pain, regardless of the task, and under the constraints of our analysis ~166.7 ms into the past, both signals from the face and heart contribute to the prediction of one another in a periodic manner (as indicated by the peaks and valleys captured in our data).

An interesting aspect of the results is that the Drawing Task, which has a higher cognitive and memory load (as it involves keeping a memory of the alphanumeric order for random spatial locations of the digits and letters across the board), showed less of an effect than the Peg Task. Both tasks included a haptic component whereby continuous pressure feedback was important. Yet the Peg Task did not have the same level of memory load or cognitive demands as the Drawing Task. In this sense, it is possible that the cognitive and memory loads acted as a distractor from the pressure pain in the Drawing Task, whereas in the Peg Task, the haptic (pressure) feedback might have amplified the pain effect. It will be important to explore this proposition with more participants, as this cohort was modest. Furthermore, it will be crucial to shuffle the tasks and perform the Drawing Task last to rule out any fatigue effects that could impact the pain outcomes. It is worth noting that the Drawing Task also showed pronounced pain effects, even though it was performed earlier than the Peg Task. The effects of pain in both tasks were highly separable. If indeed the cognitive and memory demands act as distractors from pressure pain, it will be important to further explore potential therapeutic effects of tasks that, while mediated by pressure (haptic) feedback, also have higher memory and cognitive demands in cases where descriptions of pain are verbalized as pressure-like sensations.

### 4.1 Caveats

Although our results were robust and the effects on the parameters spanning personalized families of PDFs were statistically significant, we caution that the cohort of 45 participants, with 21 included in the correlation and TE analysis, was modest. In future studies, we plan to significantly scale the study and test participants longitudinally. This would entail utilizing commercially available technologies such as smartphones and wearables that are readily accessible to the public, allowing for experimentation outside of laboratory settings. This would enable us to capture more naturalistic data and scale our research study to a larger and more diverse sample. Using these approaches can help us further ascertain the individualized (personalized) shifts in Gamma distribution families over time, as participants transition from the Control to the Pain condition. Under such conditions, we will be able to document the relevant parameter ranges across the population. These will include participants with chronic pain, transient pain, and dysregulated heart activity of various kinds, not necessarily induced by pain. For example, we have found that individuals on the autism spectrum have dysregulated heart IBI by default (Elsayed and Torres, 2023; Elsayed, 2024) during the resting state, and this dysregulated state also corresponds with dysregulated facial micro-movements at rest. We plan to explore various pathologies of the nervous system to further characterize the connection between heart and facial biorhythms.

The present analysis produced empirical families of PDFs of the moment-to-moment fluctuations present in natural human biorhythmic activities across different systems. We offer a unifying framework that characterizes and captures individualized families of distributions and parameter ranges for each person while also standardizing this process across the population. This information is crucial for generative AI models that currently rely on synthetically produced data from artificially generated noise based on theoretically assumed distributions, or mixed data from natural sets. By adopting our methods, it will be possible to generate empirically validated biological variations of time series data registered from the human nervous system. This approach can counter model autophagy disorder (MAD) (Alemohammad et al., 2023), where generative AI models degrade in performance when trained on synthetic data generated by previous iterations of the same model. There is no need for this when a single individual generates entire families of distributions capable of differentiating across tasks and different bodily and mental states (e.g., pain *vs.* pain-free states).

In conclusion, the assays employed here are simple and brief, offering new multimodal means to quantify the effects of pain experiences using our new statistical platform for individualized behavioral analysis (SPIBA) (Torres et al., 2018). This work offers new non-invasive measures, such as short video recordings of the face, to predict dysregulation of heart IBI. More erratic IBI appears to be highly predictive of corresponding facial micro-movement spikes, particularly those of the ophthalmic V1 region, where using our approach, one might be able to see more than meets the eye.

## Data Availability

The datasets presented in this study can be found in online repositories. The names of the repository/repositories and accession number(s) can be found at: https://zenodo.org/records/17082141.

## Footnotes

1
https://github.com/jlizier/jidt/
